# Epigenetic Targeting of Mcl-1 Is Synthetically Lethal with Bcl-xL/Bcl-2 Inhibition in Model Systems of Glioblastoma

**DOI:** 10.3390/cancers12082137

**Published:** 2020-08-01

**Authors:** Enyuan Shang, Trang T. T. Nguyen, Chang Shu, Mike-Andrew Westhoff, Georg Karpel-Massler, Markus D. Siegelin

**Affiliations:** 1 Department of Pathology and Cell Biology, Columbia University Medical Center, New York, NY 10032, USA; enyuan.shang@bcc.cuny.edu (E.S.); tn2387@cumc.columbia.edu (T.T.T.N.); cs485@cumc.columbia.edu (C.S.); 2 Department of Biological Sciences, Bronx Community College, City University of New York, Bronx, NY 10453, USA; 3 Department of Pediatrics and Adolescent Medicine, Ulm University Medical Center, 89081 Ulm, Germany; andrew.westhoff@uniklinik-ulm.de; 4 Department of Neurosurgery, Ulm University Medical Center, 89081 Ulm, Germany; georg.karpel@gmail.com

**Keywords:** THZ1, BH3-mimetics, Mcl1, super-enhancer, non-coding regions, epigenome

## Abstract

Apoptotic resistance remains a hallmark of glioblastoma (GBM), the most common primary brain tumor in adults, and a better understanding of this process may result in more efficient treatments. By utilizing chromatin immunoprecipitation with next-generation sequencing (CHIP-seq), we discovered that GBMs harbor a super enhancer around the Mcl-1 locus, a gene that has been known to confer cell death resistance in GBM. We utilized THZ1, a known super-enhancer blocker, and BH3-mimetics, including ABT263, WEHI-539, and ABT199. Combined treatment with BH3-mimetics and THZ1 led to synergistic growth reduction in GBM models. Reduction in cellular viability was accompanied by significant cell death induction with features of apoptosis, including disruption of mitochondrial membrane potential followed by activation of caspases. Mechanistically, THZ1 elicited a profound disruption of the Mcl-1 enhancer region, leading to a sustained suppression of Mcl-1 transcript and protein levels, respectively. Mechanism experiments suggest involvement of Mcl-1 in the cell death elicited by the combination treatment. Finally, the combination treatment of ABT263 and THZ1 resulted in enhanced growth reduction of tumors without induction of detectable toxicity in two patient-derived xenograft models of GBM in vivo. Taken together, these findings suggest that combined epigenetic targeting of Mcl-1 along with Bcl-2/Bcl-xL is potentially therapeutically feasible.

## 1. Introduction

The inherent resistance toward cell death is a challenge for glioblastoma therapy, the most common primary tumor for which no curative therapy currently exists [1,2,3]. Several types of cell death have been described, but apoptosis remains pivotal [4,5,6]. While apoptosis is regulated at several levels, the mitochondrial regulation is central and is governed by pro- and anti-apoptotic Bcl-2 family members [7,8,9]. The executer pro-apoptotic members are BAX and BAK, facilitating the liberation of cytochrome-c from the mitochondria following dissociation from their anti-apoptotic Bcl-2 family member interacting partners [9,10]. In contrast, Mcl-1 is a member of the anti-apoptotic fraction of Bcl-2 proteins, which has been shown to block therapeutic responses in glioblastoma (GBM), e.g., mediating radiation resistance [11,12]. Mcl-1 interacts with BAK and thereby interferes with the pro-apoptotic function of BAK. When Mcl-1 is inhibited, BAK is released and may facilitate cell death in concert with pro-apoptotic BAX [7,8,9,13]. Aside from Mcl-1, Bcl-2, and Bcl-xL are of interest as well. With the advent of ABT-737 in 2005, it became feasible to interfere with both Bcl-2 and Bcl-xL and thereby induce cancer cell apoptosis [7,8,9,13]. The discovery of ABT-737 followed another compound of the same class that was named ABT-263, which, in contrast to ABT-737, is orally available, rendering it clinically more amenable than its predecessor [9]. Nevertheless, researchers sought to develop the concept even further and reached another milestone with the development of the state of the art BH3-mimetic, ABT-199 (Venetoclax), a specific inhibitor of Bcl-2 with drastically lower affinity to Bcl-xL [8]. The fact that ABT-199 received FDA-approval makes it the prototype of a successful clinical translation following several decades after the original discovery of Bcl-2 in follicular lymphomas as part of the hallmark translocation between chromosome 14 and 18, which is the key diagnostic criterion of this hematological neoplasm [14,15]. However, while several tumors respond to this strategy of blocking Bcl-2/Bcl-xL, others reveal a more resistant phenotype, which in part was attributed to high levels of Mcl-1.

Targeting Mcl-1 as a treatment strategy has received recent and on-going attention. For hematological malignancies, small molecule inhibitors remain an appealing strategy, and several efforts have been launched in this regard [16]. The integration of these inhibitors is lagging behind for solid malignancies, but there may still be potential promise with early findings related to preclinical breast cancer models. However, the direct inhibition of Mcl-1 has several pitfalls [17], such as the fact that these molecules come with a high molecular weight, which, in the context of glioblastoma, might be cumbersome due to the presence of the blood brain barrier [18,19]. Therefore, alternative approaches are deemed necessary for the inhibition of Mcl-1.

Non-coding regions of the DNA have received significant attention over the last couple of years, especially in light of the fact that mutations in the coding genome are insufficient to explain the entire pathophysiology of tumors and the evolvement of treatment resistance. In this vein, the enhancer/super-enhancer landscape of tumors has been coined as a driver of tumor growth and maintenance by facilitating the up-regulation of genes in proliferation, migration, angiogenesis, and cell death [20]. Moreover, recent research clearly demonstrates that enhancers can be efficiently targeted by drug compounds that interfere with phosphorylation of RNA-polymerase II (Rpb1) [21,22]. One of these inhibitors is the CDK7/12 blocker, THZ1, that has been shown to interfere with present and emerging enhancers [21,22]. An earlier study demonstrated evidence that THZ1 exerts killing activity in GBM model system, although this was only in the context of a single treatment [23]. Here, we demonstrated that glioblastomas harbor a super-enhancer at the MCL1 locus, which translated to increase MCL1 levels as compared to normal brain tissue. While suppression of Mcl-1 alone did not yield in significant apoptosis induction, combined inhibition of Bcl-xL/Bcl-2 and Mcl-1 led to strong cell killing and a reduction of tumor growth in patient-derived xenograft models in vivo.

## 2. Results

### 2.1. Identification of a Super-Enhancer at the Mcl-1 Locus in Glioblastoma Tissues and Cells

**Figure 1:**
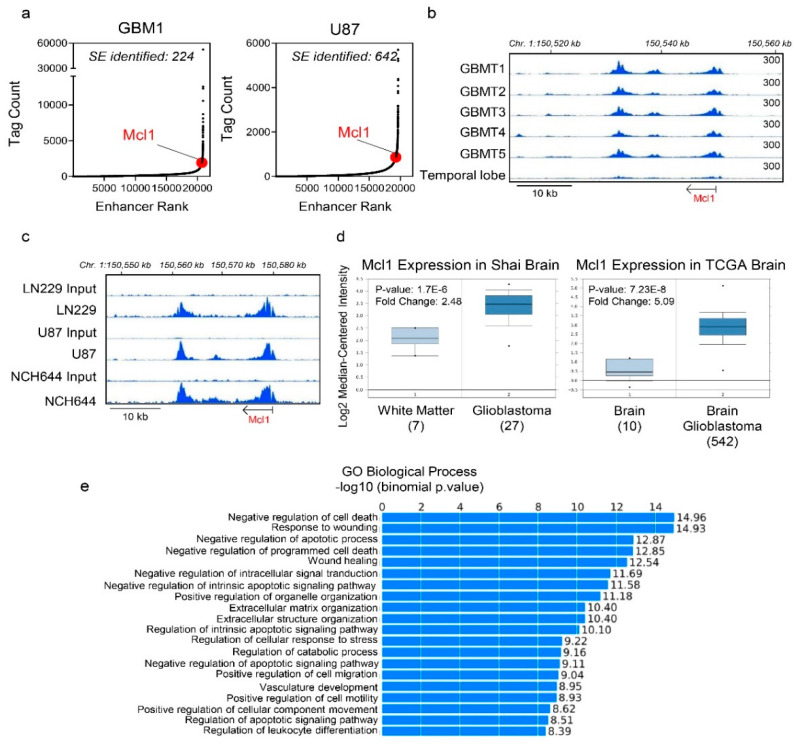
Identification of a super-enhancer related to the MCL1 gene in glioblastoma (GBM) tissues and cell cultures. (**a**) Chromatin immunoprecipitation (CHIP) with an antibody binding to H3K27ac coupled with next generation sequencing was performed on GBM1 (GBM tissue) and U87 GBM cells. Following peak calling for enhancer regions, the peaks were ranked based on the Tag count (Tag count vs. Enhancer Rank; hockey stick plot). The coordinates of the super-enhancer related to the Mcl-1 gene are chromosome 1:150,601,879-150,630,909 (GRCh38/hg38). The GBM1 chromatin immunoprecipitation with next generation sequencing (CHIP-seq) data for the super-enhancer analysis were downloaded from GEO (GSE119755); (**b**) Shown are H3K27ac CHIP-seq plots around the MCL1 locus in GBMs and normal brain tissue. The GBM tissues and the normal brain were downloaded from GEO (GSE101148; GRCh19/hg19); (**c**) Shown are H3K27ac CHIP-seq plots around the MCL1 locus in established GBM cells (LN229 and U87) and in stem-like NCH644 GBM cells; (**d**) Shown are the mRNA levels of Mcl-1 expression in Shai brain or TCGA brain databases in glioblastomas compared to normal brain tissue from oncomine (www.oncomine.org, 05/2020, Thermo Fisher, Waltham, MA, USA) [24]; (**e**) Genomic regions enrichment of annotations tool (GREAT) analysis of super enhancer genes “GO biological process.”

We interrogated publicly available datasets on GBM tissues and respective non-neoplastic brain tissue to study the epigenome related to apoptosis signaling (Figure 1a–c). Following chromatin immunoprecipitation with next generation sequencing (CHIP-seq) with the H3K27ac antibody, we studied the presence of enhancers and super-enhancers in GBM tissues and GBM cell cultures, respectively. We identified 224 and 642 super-enhancers in GBM1 and U87 cells, respectively (Figure 1a). Through this analysis and in the context of a related pathway analysis (GREAT), we identified a super-enhancer around the Mcl-1 gene in glioblastoma, which was also identified in several other GBM cultures (Figure 1a–e). It is noteworthy that, amongst the different “GO Biological Processes,” negative regulation of cell death was the most significant pathway to be modulated by the super-enhancer landscape (Figure 1e). While there are many forms of cell death (e.g., apoptosis, necrosis, ferroptosis, autophagy, etc.), it is important to note that intrinsic apoptosis emerged from this analysis, which is tightly regulated by the anti-apoptotic Bcl-2 family members of proteins. While GBM tissues displayed significant peaks around the Mcl-1 locus this phenomenon was significantly less evident in non-neoplastic brain tissue (Figure 1b). In agreement with the GBM tumors, we identified the Mcl-1 super-enhancer-related peaks in both established (U87 and LN229) and stem-like (NCH644) GBM cells (Figure 1c). Consistently, interrogation of the mRNA levels of Mcl-1 in glioblastomas and normal brain tissue confirmed that glial tumors up-regulated Mcl-1 mRNA in keeping with the emergence of a super enhancer at the Mcl-1 locus and the notion that the epigenetic regulation of Mcl-1 facilitated its increased abundance in glioblastoma as compared to normal brain tissue (Figure 1d).

### 2.2. Pharmacological Inhibition of Super-Enhancers Suppresses Mcl-1 mRNA and Protein Levels

**Figure 2:**
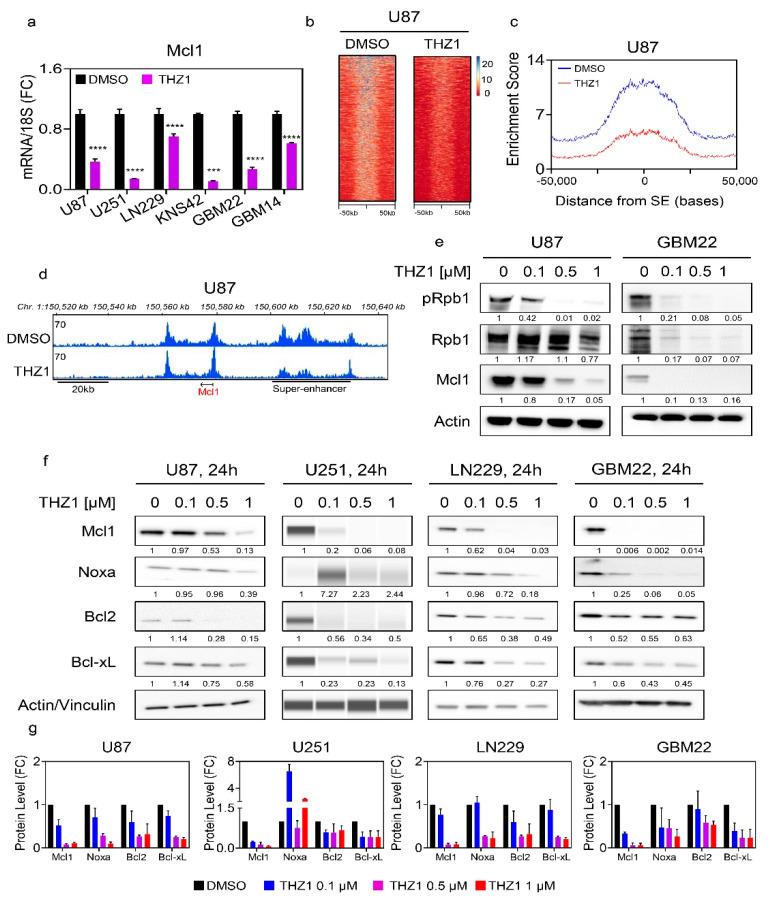
THZ1, suppresses Mcl-1 at the transcriptional level by disruption of its super-enhancer. (**a**) Real-time PCR analysis of Mcl1 mRNA levels in several GBM cells treated with vehicle or THZ1 for 24 h (*n* = 4). U87, U251, LN229, KNS42, and GBM22 cells were treated with 100 nM THZ1 while GBM14 was treated with 50 nM THZ1. Statistical significance was determined by two-tailed Student’s t-test; (**b**) U87 GBM cells were treated with DMSO or THZ1 100 nM for 24 h, subjected to CHIP with H3K27ac antibody and submitted for next generation sequencing. Super-enhancers were called using HOMER and depicted as a heatmap. The middle of each plot highlights the center of the super-enhancers (from −50 kb to 50 kb). The super enhancers are ranked by size and intensity levels are provided in the legend. The scale bar indicates the intensities. Blue depicts a high intensity level and red depicts a low intensity level; (**c**) A representation of global disruption of the super-enhancer landscape of U87 treated with DMSO or THZ1 100 nM in (**b**); (**d**) Shown are CHIP-seq (H3K27ac) tracks around the MCL1 locus (pile up values are indicated) in U87 treated with DMSO or THZ1 100 nM (super enhancer related to the Mcl-1 gene: chr1:150,601,879-150,630,909 (GRCh38/hg38)); (**e**) Standard western blots of cell lysates of U87 and GBM22 cells treated with increasing concentration of THZ1 for 24 h (pRpb1 corresponds to serine 5). Actin is used as a loading control. The protein expression levels were quantified using ImageJ (shown in cursive font); (**f**) Standard western blots or protein capillary electrophoresis of cell lysates of U87, U251, LN229, and GBM22 cells treated with increasing concentration of THZ1 for 24 h. Actin is used as a loading control in standard western blots and Vinculin is used as a loading control in protein capillary electrophoresis. The protein expression levels were quantified by using ImageJ (shown in cursive font). Uncropped blots are shown in Figure S10; (**g**) Shown are the protein expression levels of Mcl1, Noxa, Bcl2, and Bcl-xL following treatment with increasing concentration of THZ1 for 24 h in U87, U251, LN229, and GBM22 cells. FC: fold change. Shown are means and SD (*n* = 2–3). ***/**** *p* < 0.001.

Following emergence of the concept of super enhancers in cancers, the means to target them effectively were discovered, and the CDK7/12 inhibitor, THZ1, was amongst the most promising drugs [22]. To this end, we treated established and PDX GBM cultures with THZ1 and harvested mRNA to probe Mcl-1 transcripts. Given that TP53 is important in mediating apoptosis and that it is commonly mutated in gliomas, we used three established cell cultures that are either wild-type (U87) or mutated TP53 (U251 and LN229). Across all the cell lines tested, we detected a reduction of Mcl-1 transcript levels following exposure to THZ1 (Figure 2a). To confirm that these results are not only relevant to cell lines derived from adult glioblastomas, we probed the KNS-42 pediatric GBM cell line and following THZ1 treatment detected a suppression of Mcl-1 transcript levels as well (Figure 2a). We extended our studies to PDX cell cultures using the GBM22 and GBM14 lines (Figure 2a). Akin to the other cultures, THZ1 suppressed Mcl-1 transcript levels, suggesting that THZ1 appears to be a strong suppressor of Mcl-1 across a broad array of different GBM model systems.

Through the inhibition of its molecular target CDK7 and likely other targets, such as CDK12, THZ1 affects the phosphorylation of RNA-polymerase II and through this mechanism affects transcription and the modulation of cis-regulatory elements (Figure 2b–e). In keeping with this notion, we hypothesized that THZ1 should disrupt the enhancer/super-enhancer landscape of GBM cells, including the Mcl-1 super enhancer. To this purpose, U87 GBM cells were treated with DMSO or THZ1. Thereafter, chromatin was isolated and subjected to CHIP with an antibody against H3K27ac followed by next generation sequencing. Following computational analysis, we focused on the super-enhancer landscape. While we noted a strong presence of super enhancers in DMSO exposed U87 GBM cells, THZ1 potently suppressed the presence/enrichment of H3K27ac at super enhancers, in keeping with the hypothesis that THZ1 decommissioned super enhancers broadly in GBM cells (Figure 2b–d).

We compared low expressing Mcl-1 cells (astrocytes) with two GBM cells cultures (GBM22 and LN229) in the context of a CHIP-qPCR assay around the Mcl-1 locus (Figure S1a,b). In accordance with the Mcl-1 mRNA levels, we found increased enrichment in LN229 and GBM22 cells as compared to the astrocytes (Figure S1a,b). Next, we narrowed the range and focused on the genetic location around the MCL1 locus. We found that, following DMSO exposure, the enrichment of H3K27ac was highly evident, in keeping with the anatomy of an active enhancer. In contrast, we detected a disruption of the Mcl-1 super-enhancer complex following treatment with THZ1 (Figure 2d). In addition to the CHIP-seq analysis, we performed CHIP-qPCR in an established cell culture (LN229) and one PDX (GBM22) line (Figure S1c). In analogy to the experiment performed in U87 GBM cells, LN229 and GBM22 were treated with THZ1 for 24 h, harvested, and subjected to CHIP with the same antibody against H3K27ac, but in addition, we utilized an IgG control antibody as well. Following preparation, real-time PCR analysis proceeded with a primer pair that amplifies a region within the Mcl-1 locus. In both LN229 and GBM22, we detected a strong signal of H3K27ac marks within the Mcl-1 target region, whereas the IgG control yielded a significantly weaker signal, suggesting specific binding of H3K27ac at this location (Figure S1c). Importantly, THZ1 displaced H3K27ac marks from this region in both LN229 and GBM22 treated samples, in keeping with potential epigenetic disruption of the epigenetic landscape of the MCL1 locus and the CHIP-seq analysis performed in U87 GBM cells. These observations are in full agreement with the transcriptional suppression of Mcl-1 by THZ1 as shown in the real-time PCR analysis. To confirm that THZ1 is indeed acting on target, established (U87) and PDX (GBM22) GBM cells were exposed to increasing concentrations of THZ1 for 24 h. Thereafter, protein lysates were analyzed for the expression of phosphorylated Rpb1 (CDK7 and likely other targets), total Rpb1, and Mcl-1 (Figure 2e and Figure S2). Irrespective of the GBM culture tested, we detected a dose-dependent decrease of phosphorylated Rpb1 levels, which appeared to correlate with a drop of Mcl-1 protein levels (Figure 2e and Figure S2). These results are consistent with a model, proposing a direct correlation between transcriptional inhibition and concerted decline in Mcl-1 protein levels, in keeping with the mechanism of action by THZ1.

Next, we evaluated the protein levels of the most relevant anti- and pro-apoptotic Bcl-2 family members, including Mcl-1, Bcl-2, Bcl-xL, and Noxa. To this purpose, we focused again on the earlier cell cultures used for the real-time PCR analysis, ensuring a broad range of different type of tumors. All cell cultures were treated with increasing concentrations of THZ1 and, following incubation for 24 h protein, they were harvested. Thereafter, standard western blotting was employed. Independent of the TP53 status, we noted a dose dependent decrease of Mcl-1 in U251, U87, and LN229 GBM cells (Figure 2f,g). In the PDX lines, this effect was even more pronounced than in the established GBM cell cultures (Figure 2f,g and Figure S1d). Consistently, we also detected a suppression of Mcl-1 in the pediatric GBM cell line, KNS-42 (Figure S1d). These results demonstrate potent Mcl-1 protein level suppression by THZ1 across all relevant model systems tested. Mcl-1 interacts with a number of proteins, but with regard to apoptosis induction, it appears certain that Noxa is amongst the most relevant partners given that Noxa antagonizes the ability of Mcl-1 to block apoptosis by facilitating release of BAK from Mcl-1. Therefore, the ratio between Noxa and Mcl-1 is an indicator for the apoptotic threshold of tumor cells. To this end, we determined Noxa protein levels in the current cell cultures following exposure with increasing concentrations of THZ1. Across the cell cultures tested (established, PDX, and pediatric GBM cultures), we found that, in most instances, Noxa levels declined following THZ1 exposure but significantly less than Mcl-1 protein levels. We also checked the protein levels of Bcl-xL given its significant implication on the viability of solid tumors. We found that THZ1 suppressed this anti-apoptotic Bcl-2 family member as well, but in contrast to Mcl-1, much higher levels of THZ1 were necessary for the detection of this effect. We concluded with the analysis of Bcl-2 protein levels, the most prominent member within the family, and found that THZ1 also suppressed Bcl-2 protein levels (Figure 2f). It is further worth mentioning that, while the PDX lines displayed a reduction of Bcl-2 levels following THZ1 treatment, the effect on Mcl-1 appeared to be more pronounced.

### 2.3. The Super-Enhancer Blocker, THZ1, Sensitizes GBM Cells to the Cytotoxic Effects of BH3-Mimetics, and Attenuates ABT263 Mediated Up-Regulation of Mcl-1

**Figure 3:**
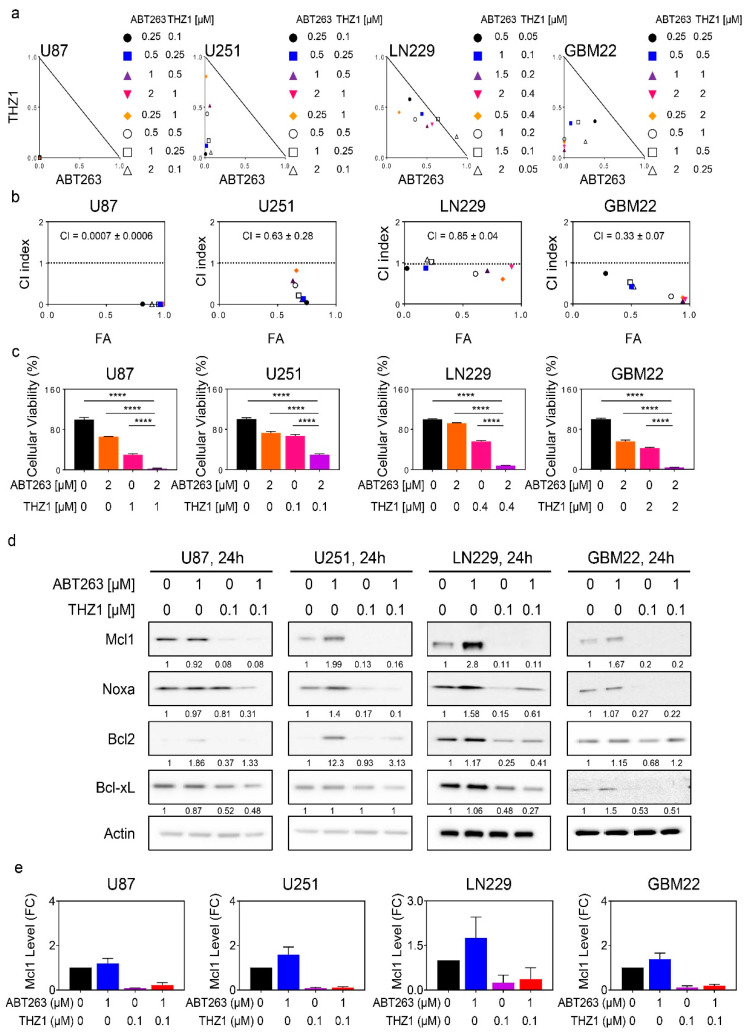
THZ1 and BH3-mimetics act synergistically to reduce the viability of glioblastoma model systems in vitro. (**a**) Cellular viability data of U87, U251, LN229, and GBM22 cells treated with ABT263, THZ1 or the combination of both. Isobolograms are shown; (**b**) Combination index (CI) value of samples treated in (**a**). CI value < 1: synergistic, CI value = 1: additive, and CI > 1: antagonistic. The dotted line represents additivity (CI value = 1). FA: fraction affected; (**c**) The graphs show cellular viability data of U87, U251, LN229, and GBM22 cells treated with ABT263, THZ1, or a combination of both (*n* = 4). ANOVA was used for statistical analysis; (**d**) Standard western blots of cell lysates of U87, U251, LN229, and GBM22 cells treated with ABT263, THZ1, or the combination of both for 24 h. Actin is used as a loading control. The protein expression levels of Mcl1, Noxa, Bcl2, and Bcl-xL were quantified by using ImageJ (shown in cursive font). Uncropped blots are shown in Figure S10; (**e**) The protein expression level of Mcl1 treated with ABT263, THZ1, or the combination of both for 24 h in U87, U251, LN229, and GBM22 cells. FC: fold change. Shown are means and SD (*n* = 2–3). ****p < 0.001.

Given the suppression of Mcl-1 by THZ1, it was tempting to speculate whether THZ1 and BH3-mimetics would induce synergistic reduction in cellular viability in GBM model system given the long list of evidence that Mcl-1 is the primary mediator of resistance for BH3-mimetics that target either Bcl-2 or Bcl-xL. To this purpose, established GBM cells (U87, U251, and LN229) were treated with ABT263, THZ1 or the combination of both. Consistent with previous observations, we noted some reduction in cellular viability by ABT263 (Figure 3a). Similarly, but slightly more pronounced, we also detected a loss in viability following THZ1 treatment. Notably, when the two compounds were combined, we detected a synergistic reduction in cellular viability (CI < 1.0). Notably, the U87 cells displayed the most significant engagement of synergistic growth reduction with an astonishing low CI value (Figure 3b). We further extended these experiments to a range of different PDX lines, including GBM22, GBM12, GBM14, GBM123, and GBM43. Akin to the established GBM cells, we noted a synergistic growth reduction when ABT263 and THZ1 were combined, which was most pronounced in GBM12 and GBM14 cells with very low CI values, mirroring the findings obtained in the U87 cells (Figure 3a–c and Figure S3a–c). Remarkably, this effect was seen at low nano-molar dosages for both ABT263 and THZ1, suggesting potential translatability. Despite this truly remarkable synergy, the GBM22 and GBM123 cells also revealed synergistic growth reduction following treatment with the combination of ABT263 and THZ1. Regarding the pediatric GBM line, we detected a synergistic growth reduction by the combination treatment in KNS-42 cells as well (Figure S3d). Finally, we asked whether stem-like GBM cells would be affected by the combination treatment because these cells have been shown to be pivotal for tumor recurrence, and killing them off may lead to a prolongation of tumor therapeutic responses. To this purpose, we embarked on NCH644 stem-like GBM cells that, from their isolation, were grown under neuro spheres conditions and have been relatively well characterized (Figure S3e). Akin to the cell lines before, we noted single-agent activity by both ABT263 and THZ1. The combination treatment resulted in significant synergistic reduction cellular viability, supporting the notion that the combination treatment is active in a broad array of GBM model systems, including therapy refractory stem-like GBM cells.

Next, we sought out to determine how the combination treatment of ABT263 and THZ1 were to affect the expression levels of the Bcl-2 family members since ABT263 is known to modulate in particular the levels of Mcl-1 and thereby causing a paradoxical partial drug-mediated resistance. Using the same panel of cell lines, we found that ABT263 increased Mcl-1 levels in U251, LN229, and GBM22 cells and that this up-regulation was suppressed by THZ1 (Figure 3d,e).

### 2.4. The Combination Treatment of ABT263 and THZ1 Elicits Enhanced Activation of a Cell Death with Features of Apoptosis

**Figure 4:**
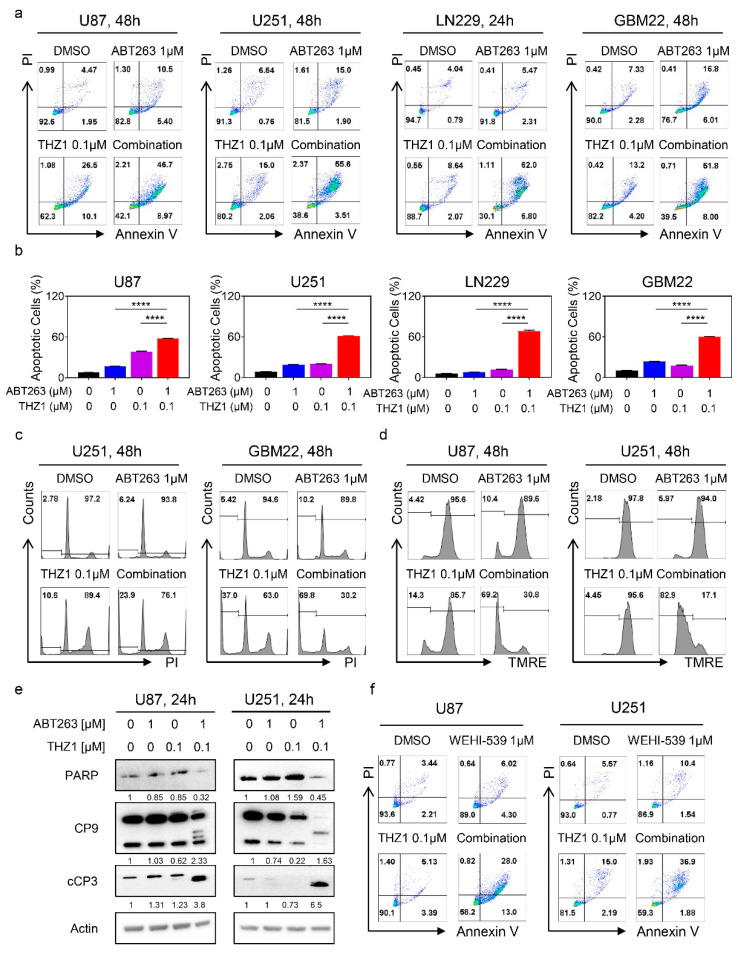
THZ1 and BH3-mimetics enhanced activation of a cell death with apoptotic features, including cleavage of initiator and effector caspases. (**a**) Representative flow plots of U87, U251, LN229, and GBM22 cells treated with ABT263, THZ1, or the combination of both for 24 h and labeled with Annexin/PI dye; (**b**) The graphs show apoptotic cells of U87, U251, LN229, and GBM22 cells treated with ABT263, THZ1, or the combination of both for 48h (*n* = 3). Shown are means and SD. ANOVA was used for statistical analysis; (**c**) Representative flow plots of U251 and GBM22 cells treated with ABT263, THZ1, or the combination of both for 24 h, fixed and labeled with propidium iodide dye; (**d**) Representative flow plots of U87 and U251 cells treated with ABT263, THZ1, or a combination of both for 24 h and labeled with TMRE dye; (**e**) Standard western blots of cell lysates of U87 and U251 cells treated with ABT263, THZ1, or a combination of both for 24 h. Actin is used as a loading control. The protein expression levels of PARP, CP9, and cCP3 were quantified using ImageJ (shown in cursive font). Uncropped blots are shown in Figure S10; (**f**) Representative flow plots of U87 and U251 cells treated with WEHI-539, THZ1, or the combination of both for 24 h and labeled with Annexin/PI dye. **** *p* < 0.001.

To accomplish the most efficient treatment against GBM, it is desirable to develop therapies that not only stall proliferation but instead induce cell death, ultimately conferring the potential to such treatments to elicit tumor regression. Given that BH3-mimetics usually elicit their anti-tumor effects by induction of apoptosis, we hypothesized that the combination treatment of ABT263 and THZ1 would lead to strong cell death induction with features of apoptosis. To this purpose, we tested established and PDX lines and treated them with ABT263, THZ1, or a combination. Following incubation, the cells were subjected to staining with annexin V/PI staining, a classical methodology to measure apoptosis. The results of the multi-parametric flow cytometry showed cell death in response to single agent therapy. However, the combination treatment was much more efficient to induce cell death, in keeping with the hypothesis that the synergistic reduction in cellular viability is likely to be largely mediated through enhanced cell death (Figure 4a,b, and Figure S4a,b). In addition to annexin V staining, we assessed DNA fragmentation by flow cytometry as well and found that the combination treatment led to enhanced increase of cells in the sub-G1 fraction, indicative of a cell death with apoptotic features (Figure 4c and Figure S4c). The classical features of intrinsic apoptosis are the loss of mitochondrial membrane potential that is succeeded by activation initiator and effector caspases followed by cleavage of caspase substrates, such as alpha-fodrin or PARP. To this end, established GBM cells (U87, U251, and LN229) were treated with ABT263, THZ1 or the combination of both and after treatment stained with TMRE (Figure 4d and Figure S5a,b). The flow cytometric analysis yielded some loss of mitochondrial membrane potential, resulting from single treatment administration. Notably, the combination treatment was much more potent than single treatments or vehicle. We confirmed these findings in PDX lines as well (Figure S5a,b). The final execution of apoptosis, be it extrinsic or in the current case intrinsic, takes place at the activation of caspases. To assess caspase activation, we utilized a well-accepted surrogate that is caspase cleavage detection by western blotting. U87 and U251 GBM cells were treated with vehicle, ABT263, THZ1, or a combination, and thereafter, protein lysates were analyzed for PARP, caspase 9, and cleaved caspase-3 levels. We found that the combination treatment led to a depletion/cleavage of PARP levels in both cell lines tested (Figure 4e and Figure S5c). We noted a depletion of total caspase 9 levels with enhanced appearance of cleavage productions of both caspase 9 and caspase 3, suggesting activation of the caspase cascade. Similar findings were obtained in LN229 GBM cells (Figure S5d).

In recent years, excitement has been generated by the emergence of selective BH3-mimetics, enabling to target just one of the Bcl-2 family members exclusively. We hypothesized that, given earlier findings, the combination treatment of BH3-mimetics and THZ1 would likely work best through Bcl-xL inhibition given its role in solid malignancies. To this purpose, U87 and U251 cells were treated with vehicle, WEHI-539 (selective Bcl-xL inhibitor) [25], THZ1, or a combination. Thereafter, cellular viability was analyzed in the context of a synergism analysis through isobologram computation, demonstrating synergistic growth reduction (Figure S6a–c). In a parallel experiment, we utilized ABT-199 (a selective Bcl-2 inhibitor) in lieu of WEHI-539. Consistently, we noted synergistic growth reduction by the combination treatment of ABT-199 and THZ1 (Figure S6d–f). Multi-parametric flow cytometric following labeling with AnnexinV/PI yielded enhanced cell killing by the combination treatment of WEHI-539 and THZ1 when compared to vehicle or single treatment (Figure 4f and Figure S6g). Next, we determined the expression levels of the Bcl-2 family of proteins following treatment with WEHI-539 and THZ1. We found that the combination treatment reduced the Mcl-1 protein levels and that the ratio between Noxa and Mcl-1 was elevated, in keeping with enhanced cell death induction. We also noted a suppression of Bcl-2 and Bcl-xL protein levels following incubation with the combination treatment (Figure S6h). Finally, we studied the effects of the combination treatment of WEHI-539 and THZ1 on caspase cleavage. Akin to the combination treatment of ABT263 and THZ1, the selective BH3-mimetic, WEHI-539, combined with THZ1 exerted significant cleavage of initiator caspase-9 and effector caspase-3 accompanied by the appearance of the 89 kDa PARP cleavage product (Figure S6i). These results suggest that selective BH3-mimetics synergize with THZ1 to induce cell death with apoptotic features, and it is encouraging to note that even ABT199 (which is FDA-approved) appeared to be effective as well.

### 2.5. Suppression of Mcl-1 by THZ1 Is Sufficient and Necessary to Sensitize GBM Cells for BH3-Mimetic-Mediated Cell Death

**Figure 5:**
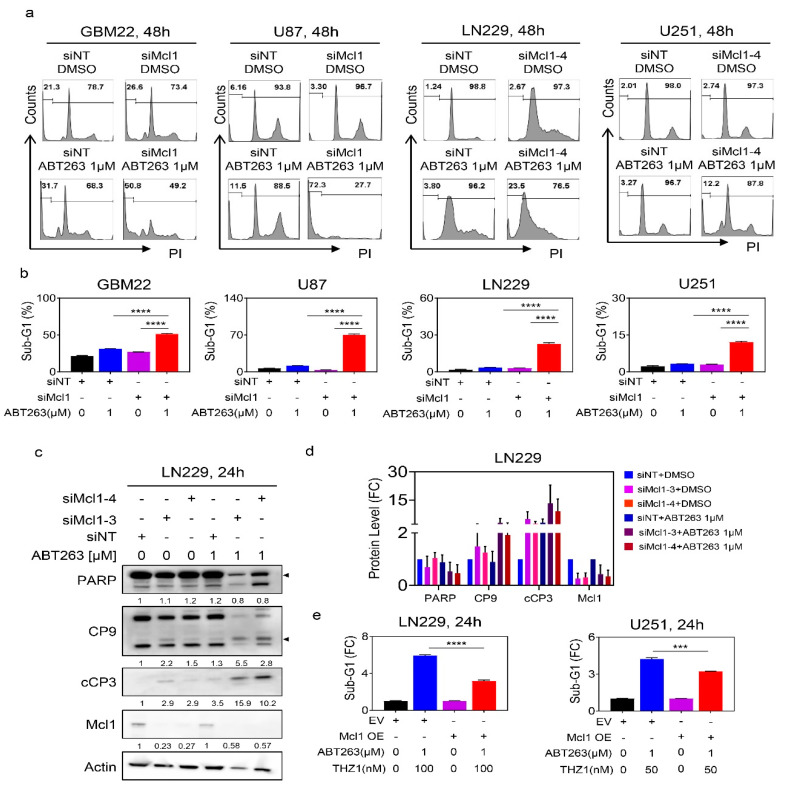
Down-regulation of Mcl-1 is necessary and sufficient for the combination treatment of ABT263 and THZ1 to exert its killing effects on GBM cells. (**a**) Shown are representative flow plots of GBM22, U87, LN229, and U251 cells transfected with control (siNT) or specific Mcl1 siRNAs, treated with ABT263, and labeled with propidium iodide followed by flow cytometry to detect the sub-G1 fraction; (**b**) The graphs show the Sub-G1 fraction of GBM22, U87, LN229, and U251 cells transfected with control (siNT) or specific Mcl1 siRNAs and subsequent treatment with vehicle or ABT263 (*n* = 3). ANOVA was used for statistical analysis; (**c**) Standard western blots of cell lysates of LN229 cells that were transfected with control (siNT) or two specific Mcl-1 siRNAs and were treated with DMSO or 1µM ABT263. The protein expression levels were quantified using ImageJ (shown in cursive font). Arrow heads show the quantification of the protein levels of total PARP and cleaved CP9. Uncropped blots are shown in Figure S10; (**d**) The protein expression levels of PARP, CP9, cCP3, and Mcl1 of LN229 cells that were transfected with control (siNT) or two specific Mcl-1 siRNAs and were treated with DMSO or 1µM ABT263 (*n* = 2–3). FC: fold change; (**e**) LN229 and U251 cells were transfected with empty vector or Mcl1 over-expressing vector and were treated with the combination of ABT263 and THZ1 for 24 h. Cells were labeled with propidium iodide and the quantifications are provided. Shown are means and SD. Statistical significance was determined by two-tailed Student’s t-test. ***/**** *p* < 0.001.

Next, we determined the role of Mcl-1 and its impact to mediate the survival of established and patient-derived GBM cultures. Despite the presence of novel BH3-mimetics that selectively target Mcl-1, we decided to utilize siRNAs to assess the impact of Mcl-1 on cell-death induction in both U87, LN229, and U251 GBM cells, representing a range of different genetic backgrounds. Following transfection with non-targeting or Mcl-1 specific siRNA and validation of knockdown, the GBM cells were subjected to treatment with vehicle or ABT263. Thereafter, the sub-G1 fraction was assessed by flow cytometric analysis (Figure 5a,b and Figure S7a). While silencing of Mcl-1 mediated a mild increase in apoptosis, ABT263 enhanced the killing effect further as compared to transfection with non-targeting siRNA, which was validated in PDX lines as well (Figure 5a,b). We went on to confirm that silencing of Mcl-1 will enhance cleavage of caspases and caspase substrates following treatment with ABT263. To this purpose, LN229 cells were transfected with non-targeting or two Mcl1-specific siRNAs. Thereafter, treatment with ABT263 was performed and the protein lysates were analyzed for the cleavage of caspases. As anticipated, silencing of Mcl-1 sensitized for ABT263 mediated cleavage of caspases and PARP in LN229 GBM cells (Figure 5c,d).

Next, we asked about the direct involvement of Mcl-1 in the reduction of cellular viability induced by ABT263 and THZ1. To this end, we harnessed a two-pronged strategy, involving both silencing and over-expression of Mcl-1. We found that in the presence of ABT263 silencing of Mcl-1 drastically reduced the viability of GBM cells and that this effect was not further enhanced when THZ1 was present, indicating that Mcl-1 is sufficient to explain how THZ1 sensitizes for BH3-mimetic mediated reduction in viability (Figure S7b). Finally, we tested whether overexpression of Mcl-1 would rescue the killing effects mediated by the combination treatment of ABT263 and THZ1. To this purpose, we transiently transfected a control plasmid or Mcl-1 cDNA into LN229 and U251 GBM cells given that these cells tolerate transient transfection reasonably well (Figure 5e). Following transfection, the GBM cells were either treated with vehicle or incubated with the combination treatment of ABT263 and THZ1. Thereafter, cells were subjected to analysis of DNA-fragmentation (sub-G1 fraction) by flow cytometry (Figure 5e). We found that over-expression of Mcl-1 cDNA partially rescued from killing by the combination treatment, suggesting that Mcl-1 is both sufficient and necessary for the combination treatment to exert its anti-glioma effects (Figure 5e). Nota bene, these findings also implicate that, while Mcl-1 is truly involved, it appears valid to conclude that downregulation of anti-apoptotic Mcl-1 is not the only factor required for the combination to drive apoptosis.

It would be of interest to elucidate the underlying mechanisms how THZ1 (a CDK7 inhibitor) would disrupt the Mcl-1 super-enhancer region, which would likely involve interference of CDK7 or potential other CDKs. To address this vital question, we utilized a structurally inactive THZ1R compound that has been chemically modified such that it no longer binds to CDK7, which, in turn, will not inhibit RNA-polymerase II activity. Therefore, it would not interfere with the super-enhancer landscape and in consequence does not affect Mcl-1 levels. To this end, we hypothesized that THZ1R would not enhance the killing effect of BH3-mimetics. We harnessed the GBM22 PDX line that was treated with vehicle, ABT263, THZ1, or THZ1R or the respective combination of ABT263 and THZ1 and ABT263 and THZ1R. While the combination treatment of ABT263 and THZ1 synergistically reduced the cellular viability, this effect was essentially abated when THZ1R was used in lieu of THZ1, suggesting that CDK7 inhibition might be important for THZ1 to enhance the killing effects of BH3-mimetics (Figure S7c). These results led us to hypothesize that the silencing of CDK7 might be sufficient to sensitize GBM cells to the cytotoxic effects of BH3-mimetics (Figure S8a–d). Silencing of CDK7 had little effect on the sub-G1 fraction of GBM22 cells as compared to transfection with non-targeting siRNA. However, knockdown of CDK7 led to some mild enhancement of ABT263-mediated increase of apoptosis (Figure S8a–d). These results suggest that the sensitization effect by THZ1 to BH3-mimetics involves additional targets other than the inhibition of CDK7.

### 2.6. The Combination Treatment of ABT263 and THZ1 Exerts Enhanced Anti-Glioma Activity in Patient-Derived Xenograft In Vivo without Significant Organ Toxicity

**Figure 6:**
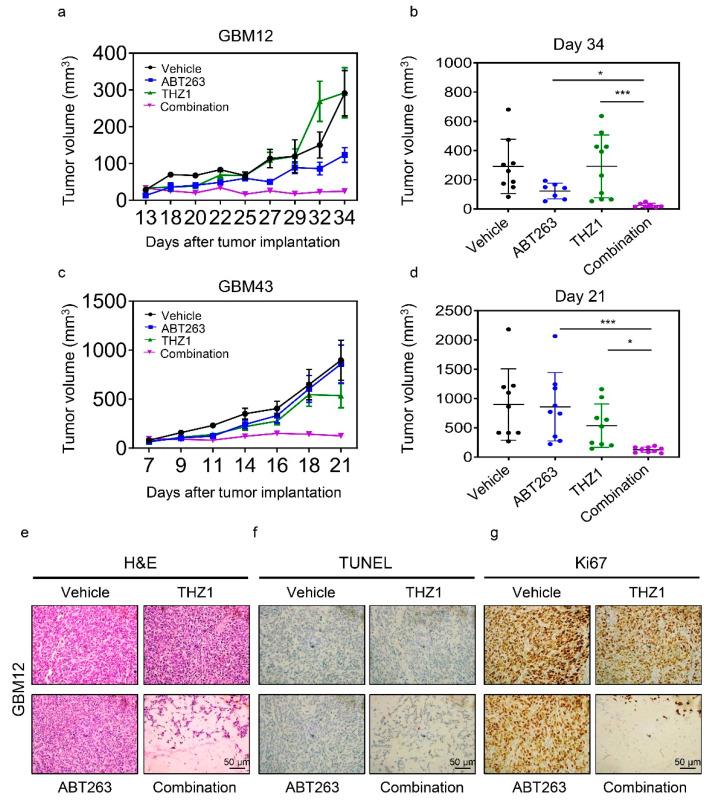
The combination treatment of ABT263 and THZ1 elicits anti-glioma activity in patient-derived xenograft model of human GBM. (**a**) GBM12 cells were implanted into the subcutis of immunocompromised Nu/Nu mice. Four randomly treatment groups—vehicle, ABT263 (75 mg/kg), THZ1 (10 mg/kg), and a combination of both—were assigned once the tumors were established. Mice were treated three times per week. Shown are the tumor volumes over time; (**b**) The graph shows tumor volume on the last day of the experiment in (**a**) (*n* = 7–10); (**c**) GBM43 cells were implanted into the subcutis of immunocompromised Nu/Nu mice. Four randomly treatment groups, vehicle, ABT263 (75 mg/kg), THZ1 (10 mg/kg), and the combination treatment of both, were assigned once the tumors were established. Mice were treated three times per week. Shown are the tumor volumes over time; (**d**) The graph shows tumor volume on the last day of the experiment in (**c**) (*n* = 9); (**e**–**g**) Tumors from the experiment in (**a**) were fixed and stained with H&E, TUNEL or Ki67. Shown are means and SD. ANOVA was used for statistical analysis. * *p* < 0.05, *** *p* < 0.001. Scale bar: 50 µm.

We utilized two PDX models system in mice to validate whether or not the combination treatment of THZ1 and BH3-mimetics would lead to enhanced reduction of tumor growth without significant toxicity, given that such successful experiments would constitute the basis for further translational development for this treatment strategy (Figure 6a–d). To this purpose, we implanted the GBM12 PDX lines in the subcutis of Nu/Nu mice. Following establishment of tumors, four groups were formed, and treatment was initiated with vehicle, ABT263, THZ1, or a combination of both. While THZ1 exerted little growth reduction in tumor growth, ABT263 reduced the size of tumors. Notably, the combination treatment of ABT263 and THZ1 led to an enhanced suppression of tumor volumes as compared to vehicle and single treatments (Figure 6a,b). To ensure that the effects are not restricted to one model system, we extended our studies to another PDX model in vivo (GBM43) (Figure 6c,d). Resembling the findings obtained in the GBM12 model, we found that the combination treatment led to a significant growth reduction of tumors as compared to single treatments. On the histopathological level, we noted that the combination treatment resulted in substantial disarray of tumor morphology with a reduction in cellular density, appearance of cell death, and a reduced occurrence of mitotic figures (Figure 6e). To further confirm the appearance of cell death, we performed TUNEL staining on the tumor slides from the various groups and noted an increase of labeled nuclei/cells in slides from tumors exposed to the combination treatment (Figure 6f). To account for the lower mitotic rate in tumors that received the combination treatment, we performed immunohistochemistry using the Ki67 antibody. We noted a substantial loss of staining in tumors treated with the combination treatment, in keeping with a reduction of mitotic figures in these specimens (Figure 6g). Given these histopathological changes on tumor tissues, it was critical to interrogate the presence of toxicity in major organ system. To this end, we performed standard H.E. staining with subsequent histopathological analysis on the brain, heart, intestine, kidney, liver, lung, and spleen (Figure S9). Our findings demonstrate viable organ systems following treatment with the combination treatment, which contrasts the histopathological findings seen in the tumor specimens.

## 3. Discussion

The landscape of the epigenome is emerging as a potential target for the therapy of cancers [26,27,28,29]. To date, there are numerous histone-modifying enzymes that can be categorized in writers, erasers, and readers. With regard to these chemical modifications, acetylation and methylation may be the most abundant ones when considering the currently available literature [30]. The differences in post translational modifications of histones establishes an epigenetic code and orchestrates the accessibility of the chromatin, thereby regulating transcription [29]. The concept of super-enhancers has recently emerged and is an interesting model to provide an epigenetic explanation as to why certain pathways are up-regulated in cancers. Enhancers are regulators of gene expression that are several kilo bases down- or up-stream of the transcriptional start site to control transcription (loop formation) and their disruption may be an emerging therapeutic opportunity [30]. Here, we found that Mcl-1 expression correlated with the presence of a super-enhancer region in glioblastoma models, providing an explanation why this protein is increased in gliomas.

The wealth of epigenetic inhibitors has enabled researchers to target the epigenome, i.e., enhancers. Considering the available literature, targeting the enhancer landscape may be accomplished by at least three different classes of inhibitors targeting bromodomain protein 4 (BRD4), histone deacetylases (HDAC), e.g., panobinostat or romidepsin, and CDK7, e.g., THZ1 [31,32]. Concerning other studies, it appears that THZ1 or recently developed derivatives are frequently used to decommission super enhancers. For instance, it has been recently shown that exposure of tumor cells to clinically validated kinase inhibitors results in reprogramming of the enhancer landscape, driving resistance to therapy [33]. This concept was exemplified in BRAF V600E mutated melanoma models exposed to kinase inhibitors, conferring immediate clinical relevance on these findings. Regarding other oncogenes, a recent intriguing observation was made related to a skull-based tumor called chordoma [34]. These neoplasms express high levels of a transcription factor, called brachyury (encoded by the TBXT gene), and critically rely on it for survival, thereby qualifying it as a marker and equally relevant as a therapeutic target. Considering the generally accepted notion that transcription factors are challenging to target, it was a welcome finding that the TBXT gene is surrounded by a super-enhancer in chordoma, thereby rendering this molecule targetable by epigenetic inhibitors, which in that study was accomplished through THZ1 [34].

Super enhancers appear to regulate the immune system, since it was recently shown that PD-L1 is encased by a super-enhancer in breast cancer cell lines [35]. Consistent with this observation, it is not surprising that a recent study found that THZ1 is capable of enhancing the efficacy of immune therapy in model systems of lung cancer [36]. Our research findings complement these earlier studies in that we utilized THZ1 to disrupt the Mcl-1 super enhancer. By conducting CHIP-seq (H3K27ac) in vehicle and THZ1-treated GBM cells, we confirmed that the Mcl-1 super-enhancer was indeed disrupted by THZ1. In turn, THZ1 potently suppressed Mcl-1 transcript and protein levels, respectively. As expected, this was accompanied by reduced phosphorylation of RNA-polymerase II. In agreement with prior publications, we noted a total reduction of RNA-polymerase II following THZ1 treatment as well [37,38]. It is noteworthy that only a moderate amount of cell death was induced by THZ1 in keeping with our notion that silencing of Mcl-1 itself is not particularly efficacious to induce cell death in our model systems tested. However, other model systems have shown different responses to THZ1, such as certain non-solid malignancies [37]. We recognized that THZ1 also down-regulated the protein levels of Bcl-xL, but as stated, much higher concentrations of THZ1 were necessary. The most likely explanation of this phenomenon may be due to the fact that Mcl-1 has a rather short half-life [7,39,40,41], and therefore, changes in expression may become apparent much quicker as compared to proteins that are more stable. We also acknowledge that likely additional targets other than CDK7 are involved in the effects elucidated by THZ1, e.g., such as CDK12.

Given the limited cell death induction by THZ1, we asked whether the killing efficacy of this compound might be enhanced in the presence of BH3-mimetics, such as ABT263. This hypothesis largely emerged from the fact that THZ1 suppressed Mcl-1 protein levels and that drugs that lower Mcl-1 levels have commonly been associated to enhance the efficacy of BH3-mimetics that target either Bcl-2, Bcl-xL, or both [7,9,42,43,44,45,46]. Consistently, we found that THZ1 and BH3-mimetics synergistically reduced the viability of GBM cells across a broad range of different model systems of human GBM. Our findings are in line with earlier ones obtained by another group. They reported that ABT263 and THZ1 reduced the viability of cholangiocarcinoma cells in a synergistic manner [47]. Interestingly, forced expression of Mcl-1 partially counteracted the killing effect by the combination, establishing the overall concept that Mcl-1 is sufficient and necessary for the combination treatment to exert its effects in GBM cells. Our focus mostly rested on the BH3-mimetic ABT263 given its inhibitory effect on Bcl-xL, since Bcl-xL has been shown to be relevant for counteracting apoptosis, especially in solid malignancies [17]. Consistently, the BH3-mimetic, ABT199, was slightly less efficient in line with its target, Bcl-2 that has been coined to be not as relevant for the survival of solid tumors as compared to Bcl-xL. Whether for instance selective Mcl-1 inhibitors would synergize with THZ1 is an interesting question, but given that our focus rested on the ability of THZ1 to suppress Mcl-1, we did not address this question. For instance, it may be possible that, given that THZ1 down-regulates Mcl-1 and thereby depletes the actual drug target, the inhibitor may become less effective.

Translational cancer research requires the critical in vivo assessment of drug compounds, given that in vitro results are insufficient for a valid conclusion as to whether or not a certain treatment may show promise as a novel therapy in cancer [48,49]. We assessed the combination treatment of BH3-mimetics and THZ1 in heterotopic PDX models in mice and found that the combination treatment exerted enhanced tumor growth when compared to single treatments without induction of toxicity. A limitation of the current work is that our studies did not include orthotopic in vivo studies, given that the microenvironment and the blood brain barrier might interfere with the efficacy of the treatment. These results are in keeping with earlier drug combinations involving BH3-mimetics [7,9,42,43,44,45,46].

## 4. Materials and Methods

### 4.1. Cell Cultures and Growth Conditions

GBM22, GBM123, GBM14, and GBM12 were obtained from the Mayo Clinic Brain Tumor Patient-Derived Xenograft National Resource (Mayo Clinic, Rochester, MN, USA). U251 cell line was obtained from Sigma (St. Louis, MO, USA). U87 and LN229 were purchased from the American Type Culture Collection (Manassas, VA, USA). KNS42 was purchased from Riken BioResource Research Center (Ibaraki, Japan). Cells were cultured in DMEM medium (Fisher Scientific (Waltham, MA, USA), MT10013CV) containing 10% FBS (Gemini, NY, USA, FBS002) and 100 μg/mL of primocin (Invivogen, CA, USA, ant-pm-1). For the experimental conditions, cells were cultured in DMEM containing 1.5% FBS and primocin. NCH644 stem-like glioma cells were obtained from Cell Line Services (CLS, Heidelberg, Germany) and were cultured in GBM-MG from Cell Line Services (CLS, Heidelberg, Germany, 820403) for both maintenance and experiments. All cell lines were incubated at 37 °C in an atmosphere containing 5% CO_2_.

### 4.2. Reagents

ABT-263 (S1001) and THZ1 (S7549) were purchased from Selleckchem (Houston, TX, USA). WEHI-539 (A3935) and ABT-199 (A8194) were purchased from APexBio Technology (Houston, TX, USA). All chemicals were dissolved in DMSO to yield a 10 mM stock solution.

### 4.3. Real-Time PCR Analysis

Cells were harvested and extracted mRNA using the miRNAeasy Mini Kit (QIAGEN, Germantown, MD, USA, 217004) following the company’s instructions. After extracting mRNA, reverse-transcription (cDNA synthesis kit (Quantabio 101414-106), and real time PCR (SYBR green RT-PCR reagents kit (Quantabio, Beverly, MA, USA, 101414-276) were used. The condition for the real-time PCR machine (Quantabio) is as follows: 95 °C for 10 min, 40 cycles of 95 °C for 15 s, 60 °C for 30 s, and 72 °C for 30 s. The fold changes were calculated based on 18S in the threshold cycle (Cq). The sequence primers are shown in Table 1.

### 4.4. Chromatin Immunoprecipitation (CHIP) Sequencing

Chromatin immunoprecipitation (CHIP) of DMSO treated and THZ1 treated samples were performed with H3K27ac (CST 4535, 10 μL/sample) or Rabbit IgG (CST 2729, 2 μL/sample) following the company’s instructions (SimpleChIP^®^ Enzymatic Chromatin IP KiT (Magnetic Beads), Cell Signaling Technology (CST), Danvers, MA, USA, 9003). ChIP DNA was assessed for quality by real-time PCR analysis before sequencing. After next-generation sequencing library preparation, the samples were run on an Illumina HiSeQ instrument (HiSeq 4000 (Genewiz, NJ, USA); single read 50 bp (SR50)).

### 4.5. Computational Analysis of CHIP Sequencing Data

The fastq files were aligned to the human genome (hg38) with bowtie2 followed by peak calling and generation of bedgraph files, utilizing the MACS2 tool [50]. The bedgraph files were converted to bigwig files to display them in the integrated genome browser (IGB). For the generation of heatmaps and plot profiles, we used deeptools [51]. To generate the matrix for heat map generation, computematrix was used, and the output options were assigned as reference-point based. The heat maps and the graphical representation of super-enhancers were generated with plotheatmap and plotprofile, respectively. Regarding the enhancer analysis, we employed HOMER-software (v4.11, 10-24-2019) [52], which relies on a modified strategy derived from ranking of super-enhancers (ROSE). In short, larger peaks were stitched together within a distance of 12.5 kb. These regions are then scored and ranked. All enhancers that lie above the defined threshold slope of 1.0 are defined as super enhancers. Super enhancers were functionally analyzed through the genomic regions enrichment of annotations tool (GREAT) and assigned GO-terms related to the biological processes most likely governed by these cis-regulatory elements. The raw and processed files (U87 treated with DMSO or THZ1 for 24 h) were deposited in GEO (GSE150986) (this study). The underlying data for the super-enhancer analysis shown in Figure 1a was downloaded from GEO (GSE119755). The underlying data for GBM tissues and the normal brain were downloaded from GEO (GSE101148).

### 4.6. Cell Viability Assays

The determination of cellular viability of the various GBM cells occurred in 96-well plates following treatments. We used a luminescence method (CellTiter-Glo^®^ assays) in accordance with instructions manual (Promega (Madison, WI, USA), G7571). To determine drug synergism the median-effect equation (Chou-–Talalay) was employed (isobolograms and the combination index (CI)) [53].

### 4.7. Flow Cytometry

Following the various treatments of GBM cell cultures, staining was performed in accordance with the manufacturers’ instructions. Annexin V Apoptosis Detection Kit (BD Pharmingen (Franklin Lakes, NJ, USA), BD 556419) was used to detect apoptosis. Propidium Iodide (PI)/RNase Staining Solution was employed (Cell Signaling Technology (Danvers, MA, USA), CST 4087S) to detect the subG1 fraction. TMRE staining (Mitochondrial Membrane Potential kit (CST 13296S)) was conducted to detect mitochondrial membrane potential. Collected data was analyzed by FlowJo software (version 10.6.2; Tree Star, Ashland, OR, USA).

### 4.8. Transfections of siRNAs and cDNA Plasmid Constructs

For overexpressed experiment, cells were transfected using Lipofectamine 3000 (Invitrogen, San Diego, CA, USA) and harvested after 24 h after transfection. pcDNA-3 and pcDNA3-hMcl1 (Addgene 25375) were purchased from Addgene (Watertown, MA, USA). For knocked down experiment, cells were transfected by using Lipofectamine RNAiMAX (Invitrogen (Carlsbad, CA, USA), 13778075) for 72 h in accordance with the manufacturers’ instructions. Non-Targeting siRNA-pool (D-001810-10-20), siMcl1 pool (L-004501-00-0005), siMcl1-3 (J-004501-16-0002), siMcl1-4 (J-004501-17-0002), and siCDK7 pool (L-003241-00-0005) were purchased from Dharmacon (Lafayette, CO, USA).

### 4.9. Western Blot Analysis

Cells were lysed in the laemmli buffer (Biorad) containing 1X protease and phosphatase inhibitor cocktail (Thermo Fisher, Waltham, MA, USA, 78440). Cell lysates were run on a 4–12% SDS PAGE gel (Invitrogen NP0321BOX), the proteins were transferred to a PVDF membrane, and the membrane was blocked with 5% skim milk (VWR (Bridgeport, NJ, USA) 97063-958) in TBST (0.1% Tween20) and probed with target antibodies. Primary antibody incubations were performed overnight at 4 °C. Antibodies were used Rpb1 (CST 14958), p-Rpb1 (CST 4735), PARP (CST 9532; 1:500), cCP9 (CST 7237; 1:500), cCP3 (CST 9665; 1:500), Mcl-1 (CST 5453; 1:500), Bcl-xL (CST 2764; 1:500), Bcl-2 (CST 4223; 1:500), Noxa (Calbiochem (Burlington, MA, USA) OP180, clone 114C307; 1:500), β-actin (Sigma Aldrich A1978, clone AC15; 1:2000). The HRP linked secondary antibodies were from Santa Cruz Biotechnology Inc. The target protein was detected on the Azure (C300) imaging system. In selected cases, the protein capillary electrophoresis (Protein Simple (San Jose, CA, USA) SM-W004) was used. The following antibodies were applied Mcl-1 (CST 5453; 1:25), Bcl-xL (CST 2764; 1:25), Bcl-2 (R&D System, MN, USA, MAB827; 1:25), Noxa (Calbiochem OP180, clone 114C307; 1:25), and Vinculin (Abcam (Cambridge, MA, USA) ab129002, 1:500).

### 4.10. Subcutaneous Xenograft Model

GBM12 and GBM43 glioblastoma patient-derived xenograft (PDX) tumors were injected into the flanks of 6–8-week-old Nu/Nu mice. After tumors were formed, the mice were randomly assigned into four treated groups: vehicle, ABT-263 (75 mg/kg), THZ1 (10 mg/kg), or ABT-263 and THZ1. Drugs were dissolved in DMSO, Kolliphor EL (Sigma, 61791-12-6), Ethyl Alcohol 200 Proof (Pharmco-Aaper, 64-17-5) and PBS at the following ratio: 10:32:8:50 (*v/v/v/v*). Tumor and weight measurements were performed three times a week, followed by intraperitoneal administration of the compounds. The length and width of tumors were calculated based on the formula: (length × width^2^)/2.

### 4.11. TUNEL and Ki67 Staining

The tumors were fixed in 4% formaldehyde (Thermo Fisher 28908) for 24 h and embedded in paraffin. Following dewaxing and rehydration, the tumors were exposed to proteinase K (Agilent Dako, S3020). To detect apoptosis, the processed slides were exposed to TUNEL reaction mixture and the reaction was terminated in the converter peroxidase solution. The chromogen, diaminobenzidine, was employed for visualization of the TUNEL-reaction. Background nuclear staining was established with hematoxylin. To detect cell proliferation, the processed slides were incubated with Ki67 antibody (Agilent Dako, GA626).

### 4.12. Statistical Analysis

Statistical significance was assessed by Student’s t-test or ANOVA (for multiple comparison) using Prism 8 (GraphPad, La Jolla, CA, USA). A *p* ≤ 0.05 was set as the level of statistical significance. * *p* < 0.05, ** *p* < 0.01, ***/**** *p* < 0.001, n. s. indicates not significant.

### 4.13. Study Approval

All procedures were in accordance with Animal Welfare Regulations and approved by the Institutional Animal Care and Use Committee at the Columbia University Medical Center (AC-AABC6505).

## 5. Conclusions

Based on our findings, we conclude that non-coding DNA elements are involved in mediating resistance to intrinsic apoptosis in model system of human GBM, which is exemplified by the identification of a super-enhancer, spanning the MCL1 locus. Disruption of the Mcl-1 super enhancer through THZ1 sensitizes a broad array of GBM cells to the cytotoxic effects by broad and selective BH3-mimetics in vitro and in vivo. These findings also bear translational relevance given that both CDK7 inhibitors and BH3-mimetics have either reached clinical testing or are even FDA-approved.

## Figures and Tables

**Table 1:** Primers for real-time PCR.

Gene	Forward Sequence	Reverse Sequence
18S	AGTCCCTGCCCTTTGTACACA	GATCCGAGGGCCTCACTAAAC
Mcl1	CCAAGAAAGCTGCATCGAACCAT	CAGCACATTCCTGATGCCACCT
ChIP-Mcl1-R1	ATGTCGCCCGAAGAGGAGCTGGACG	CAGCGACTGCCGGTACAACTCGTCC

## Supplementary Materials

The following are available online at https://www.mdpi.com/2072-6694/12/8/2137/s1, Figure S1. THZ1 inhibits the presence of H3K27ac histone marks at the MCL1 locus and suppresses Mcl-1 protein levels in GBM PDX cell cultures.; Figure S2. THZ1 affects the levels of phosphorylated RNA-polymerase II (Ser5), total RNA-polymerase and Mcl-1 protein levels.; Figure S3. THZ1 and BH3-mimetics act synergistically to reduce the viability of glioblastoma model systems in vitro.; Figure S4. THZ1 and BH3-mimetics combination treatment leads to an enhancement of cell death.; Figure S5. The combination treatment of THZ1 and BH3-mimetics leads to an enhancement of cell death with apoptotic features.; Figure S6. Selective BH3-mimetics synergize with THZ1 to induce cell death with apoptotic features.; Figure S7. Down-regulation of Mcl-1 is involved in the combination treatment of ABT263 and THZ1 to exert its killing effects on GBM cells.; Figure S8. The sensitization effect by THZ1 to BH3-mimetic-mediated cell death involves additional targets other than the inhibition of CDK7.; Figure S9. The combination treatment of ABT263 and THZ1 enhanced anti-glioma activity in patient-derived xenograft in vivo without organ toxicity; Figure S10. Uncropped Western Blot Figures.
