# Supplementary material for: Epigenetic Targeting of Mcl-1 Is Synthetically Lethal with Bcl-xL/Bcl-2 Inhibition in Model Systems of Glioblastoma
Source: Cancers (Basel). 2020 Aug 1;12(8):2137. doi: 10.3390/cancers12082137 (PMC7464325; doi:10.3390/cancers12082137)
Supplement: Supplementary file 1 [file cancers-12-02137-s001.zip › cancers-859599-supplementary final/cancers-859599 - supplementary layout S1-9.docx]

Supplementary Materials

Epigenetic Targeting of Mcl-1 is Synthetically Lethal with Bcl-xL/Bcl-2 Inhibition in Model Systems of Glioblastoma

Enyuan Shang ^1,2,†^, Trang T. T. Nguyen ^1,†^, Chang Shu ^1^, Mike-Andrew Westhoff ^3^, Georg Karpel-Massler ^4^ and Markus D. Siegelin ^1^


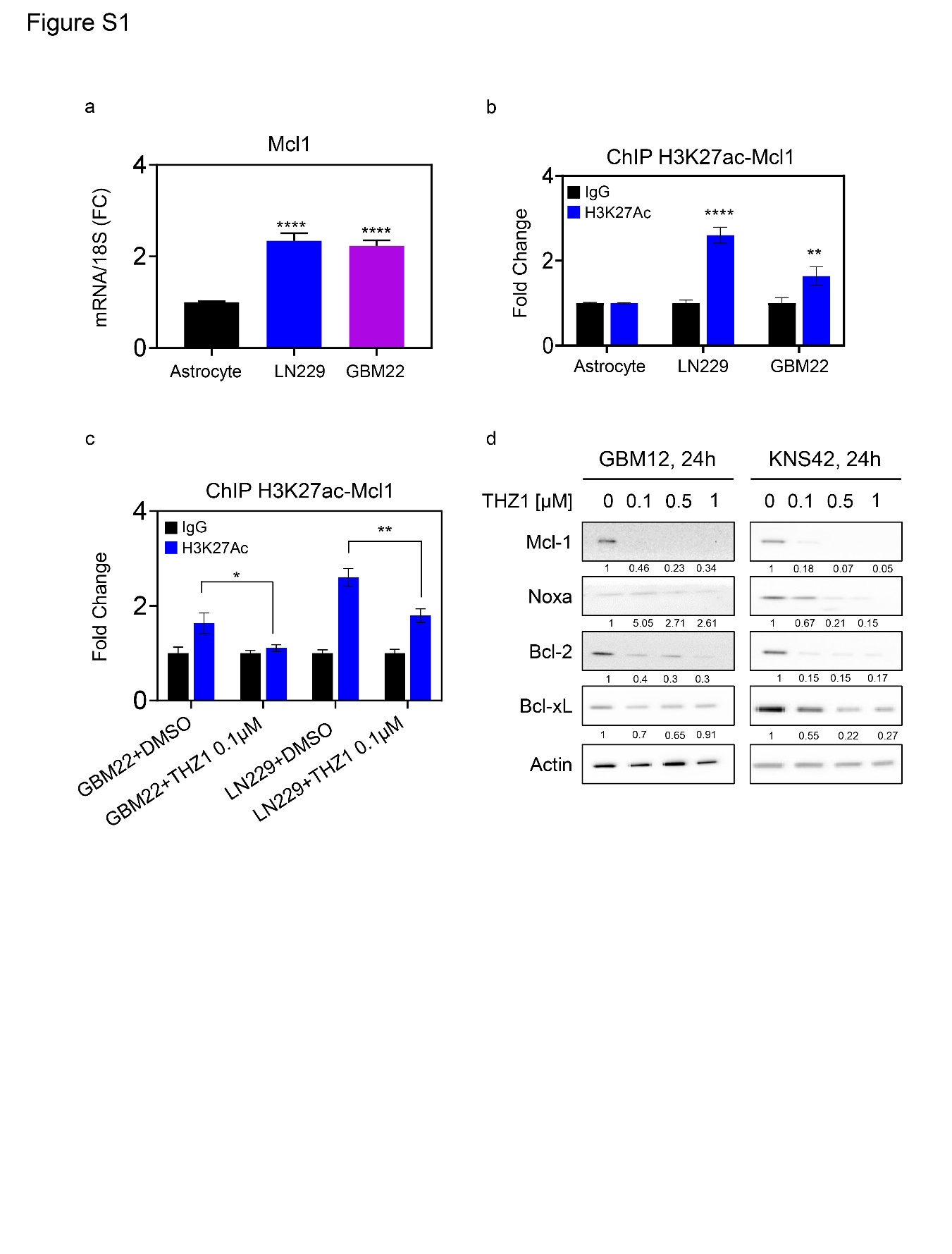


**Figure S1.** THZ1 inhibits the presence of H3K27ac histone marks at the MCL1 locus and suppresses Mcl-1 protein levels in GBM PDX cell cultures. (**a**) Real-time PCR analysis of Mcl1 mRNA levels in astrocytes, LN229, and GBM22 (*n* = 4); (**b**) ChIP-qPCR (with H3K27ac antibody) of the MCL1 locus of astrocytes, GBM22, and LN229 cells (*n* = 3). Shown are means and SD. ANOVA was used for statistical analysis; (**c**) ChIP-qPCR (with H3K27ac antibody) of the MCL1 locus of GBM22 and LN229 GBM cells treated with DMSO or 100 nM THZ1 for 24 h (*n* = 3). Shown are means and SD. Statistical significance was determined by two-tailed Student’s t-test; (**d**) Standard western blots of cell lysates of GBM12 and KNS42 cells treated with DMSO or increasing concentration of THZ1 for 24h. Actin is used as a loading control. The protein expression levels of Mcl1, Noxa, Bcl2, and Bcl-xL were quantified by using ImageJ (shown in cursive font). Uncropped blots are shown in Figure S10. **p* < 0.05, ***p* < 0.01, *****p* < 0.001.


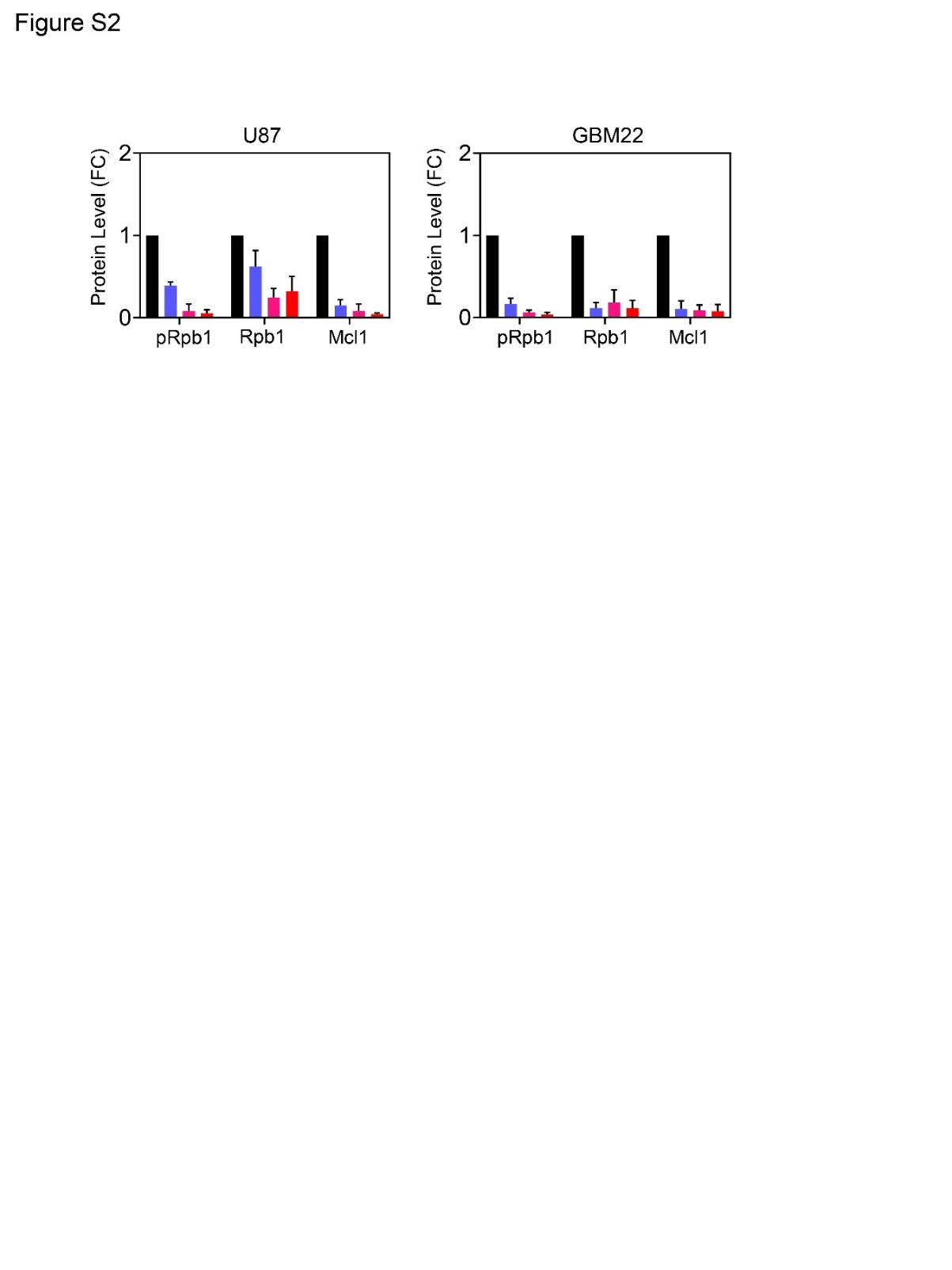


**Figure S2.** THZ1 affects the levels of phosphorylated RNA-polymerase II (Ser5), total RNA-polymerase II and Mcl-1 protein levels. The protein expression levels of pRpb1, Rpb1, and Mcl1 treated with increasing concentration of THZ1 in U87 and GBM22 cells for 24 h are shown. FC: fold change. Shown are means and SD (*n* = 2–3). Black bar: DMSO, blue bar: THZ1 0.1 µM, pink bar: THZ1 0.5 µM, and red bar: THZ1 1 µM.

**
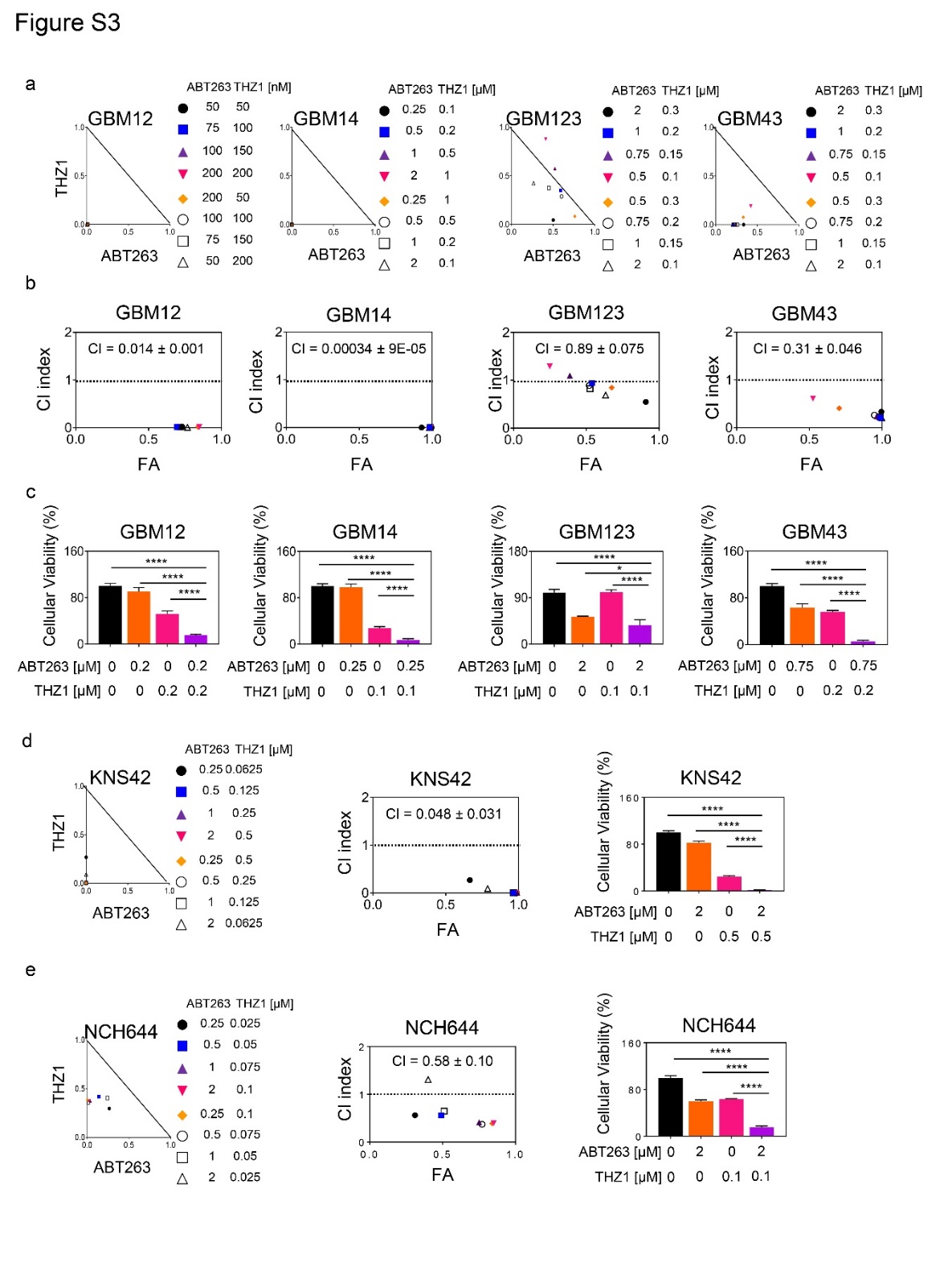
**

**Figure S3.** THZ1 and BH3-mimetics act synergistically to reduce the viability of glioblastoma model systems in vitro. (**a**) Isobolograms of GBM12, GBM14, GBM123, and GBM43 cells treated with ABT263, THZ1 or the combination of both; (**b**) CI (Combination Index) value of samples treated in (**a**). CI value < 1: synergistic, CI value = 1: additive, and CI > 1: antagonistic. The dotted line represents additivity (CI value = 1). FA: fraction affected; (**c**) The graphs show the cellular viability of GBM12, GBM14, GBM123, and GBM43 cells treated with ABT263, THZ1, or combination of both (*n* = 4); (**d)** Shown are an isobologram, a combination index graph, and a graph depicting cellular viability of KNS42 cells treated with the indicated concentrations of ABT263, THZ1 or the combination; (**e**) Shown are an isobologram, a combination index graph, and a graph depicting cellular viability of NCH644 cells treated with the indicated concentrations of ABT263, THZ1 or the combination of both. Shown are means and SD. ANOVA was used for statistical analysis. **p* < 0.05, *****p* < 0.001.


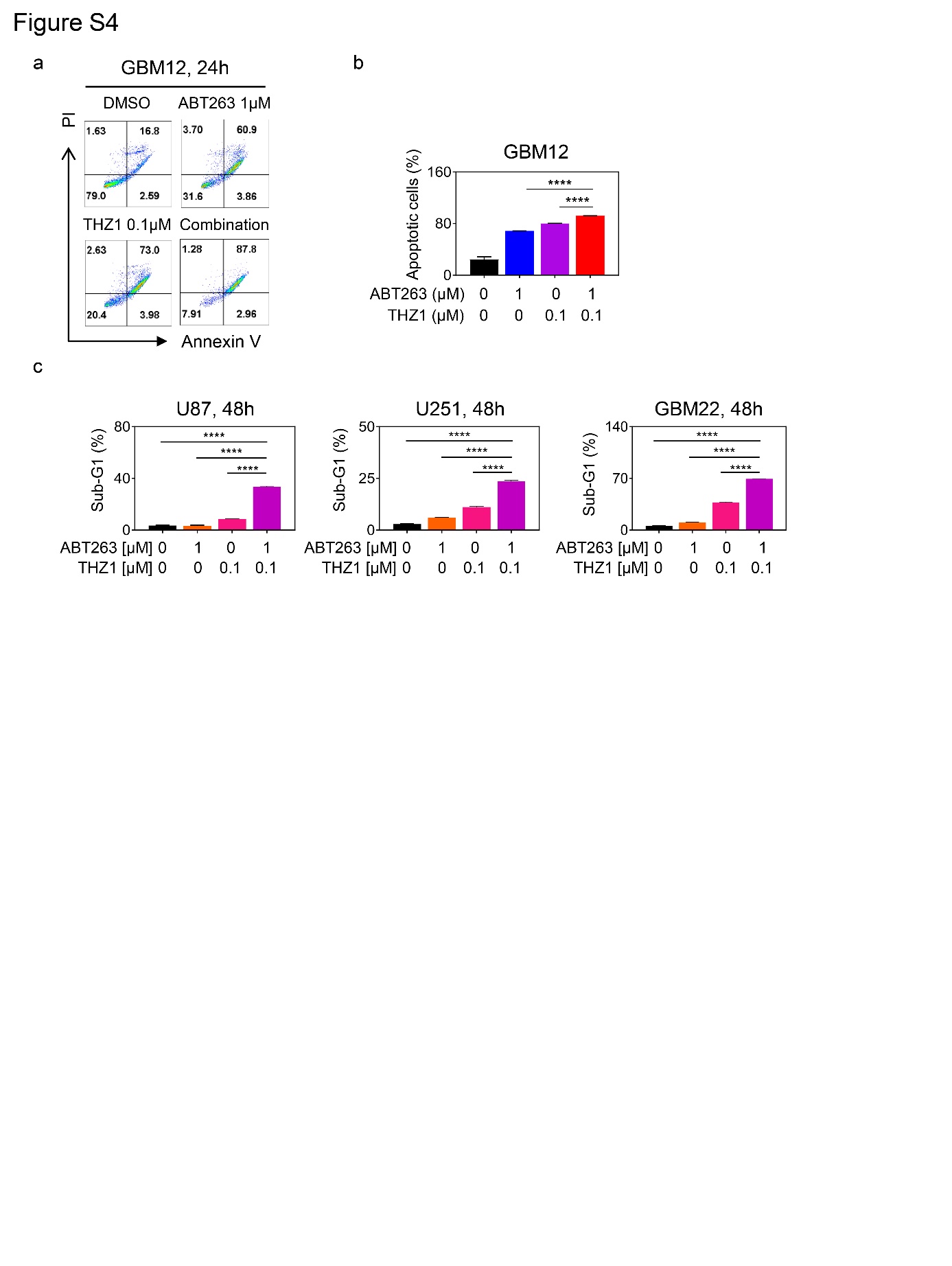


**Figure S4.** THZ1 and BH3-mimetics combination treatment leads to an enhancement of cell death. (**a**) Shown are representative flow plots of GBM12 cells treated with ABT263, THZ1, or the combination of both that were labeled with Annexin/PI dye; (**b)** The graph shows apoptotic cells of ABT263, THZ1, or combination of both in GBM12 cells (*n* = 3); (**c**) The graphs show sub-G1 cells of ABT263, THZ1, or combination of both in U87, U251, and GBM22 (*n* = 3). Shown are means and SD. ANOVA was used for statistical analysis. *****p* < 0.001.

**
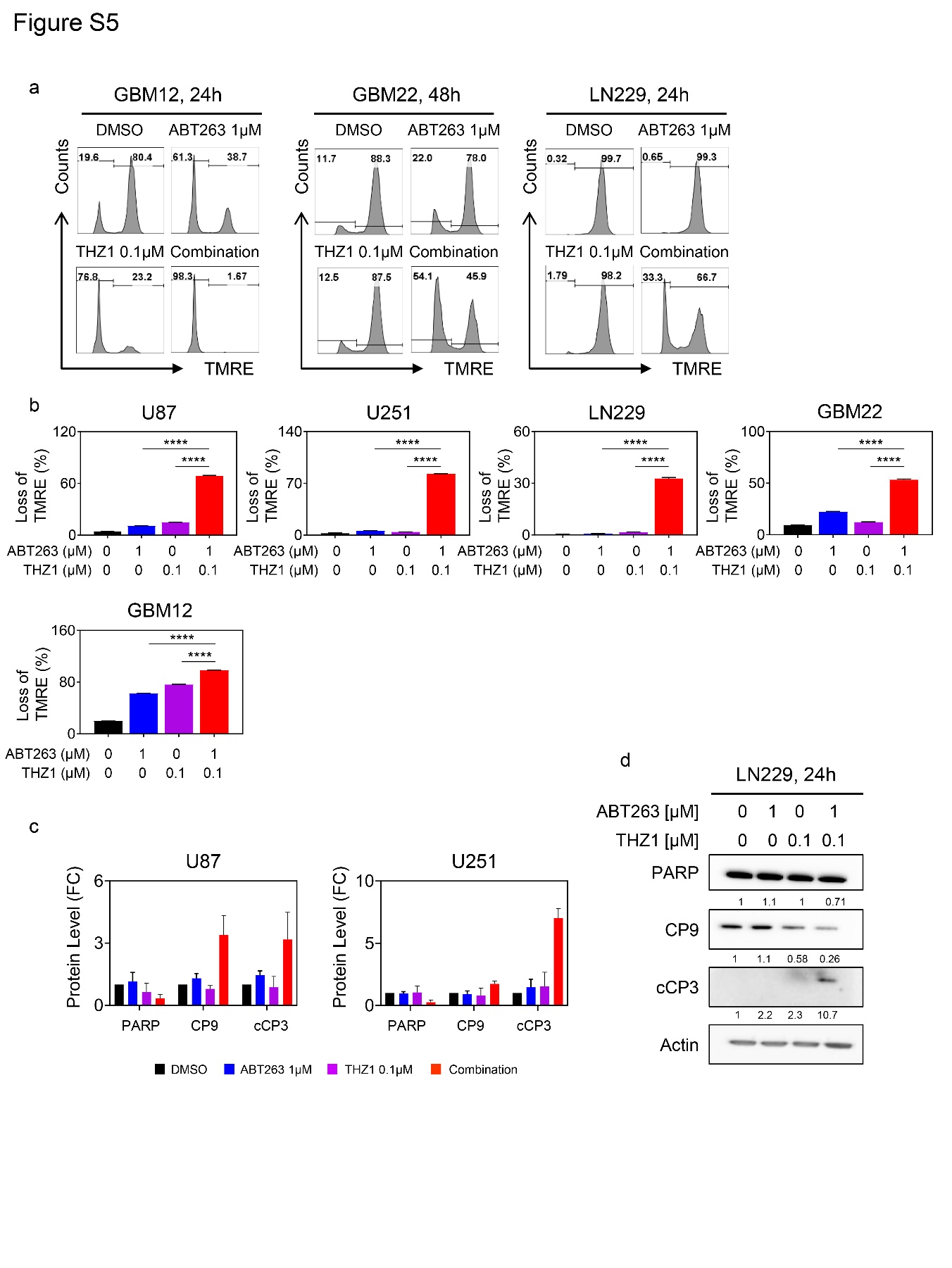
**

**Figure S5.** The combination treatment of THZ1 and BH3-mimetics leads to an enhancement of cell death with apoptotic features. (**a**) Shown are representative flow plots of GBM12, GBM22, and LN229 cells treated with ABT263, THZ1, or the combination of both and labeled with TMRE dye; (**b**) The graphs show loss of TMRE of ABT263, THZ1, or combination of both for 48 h in U87, U251, LN229, GBM22, and GBM12 cells (*n* = 3). Shown are means and SD. ANOVA was used for statistical analysis; (**c**) Shown are the protein expression levels of PARP, CP9, and cCP3 treated with ABT263, THZ1, or combination of both in U87 and U251 cells. FC: fold change. Shown are means and SD (**n** = 2–3); (**d**) Standard western blots of cell lysates obtained from LN229 cells treated with ABT263, THZ1 or the combination of both for 24 h. Actin serves as a loading control. The protein expression levels of PARP, CP9, and cCP3 were quantified by using ImageJ (shown in cursive font). Uncropped blots are shown in Figure S10. *****p* < 0.001.

**
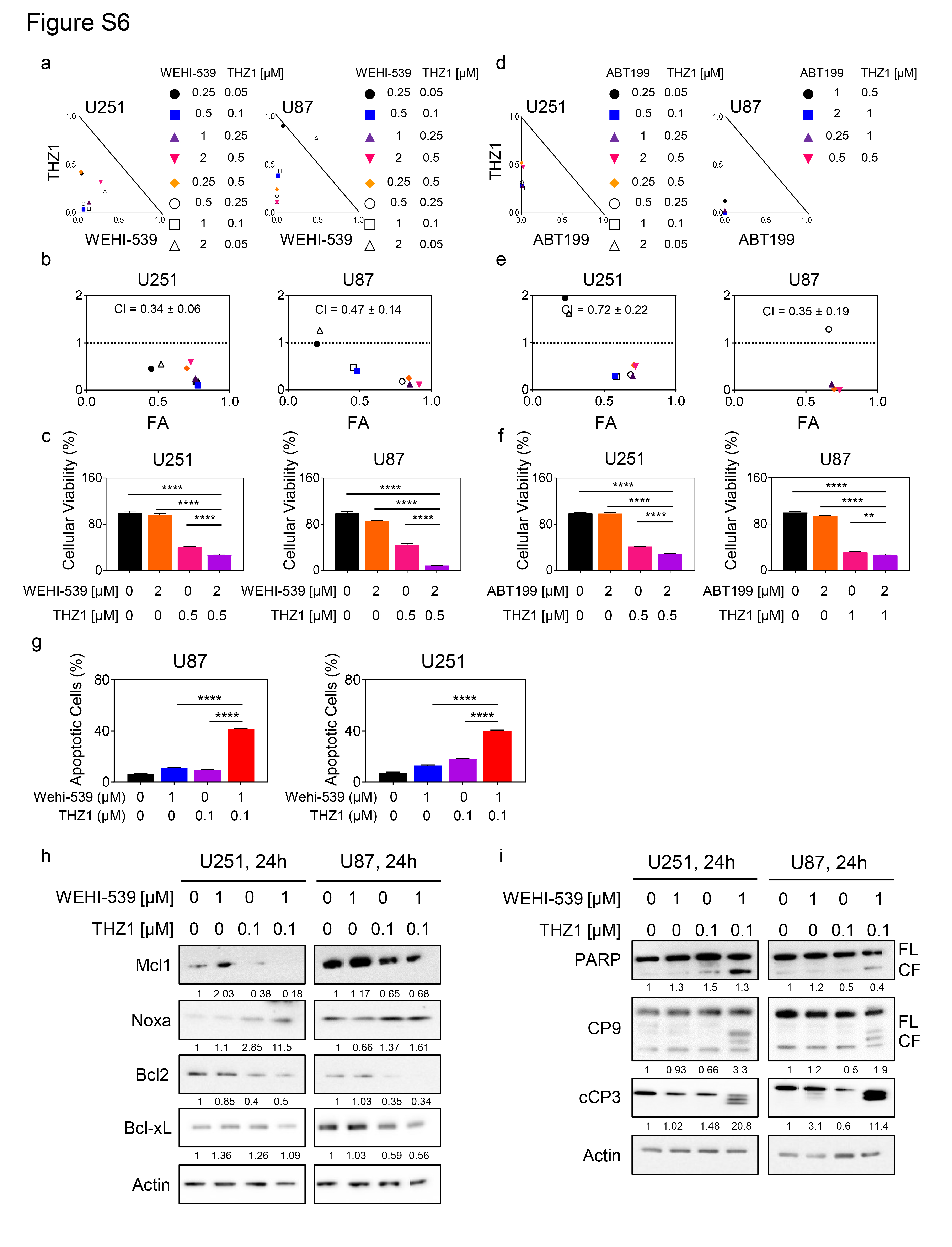
**

**Figure S6.** Selective BH3-mimetics synergize with THZ1 to induce cell death with apoptotic features. (**a**) Shown are the isobolograms of U251 and U87 cells treated with WEHI-539, THZ1 or the combination of both; (**b**) Shown are graphs displaying the CI (Combination Index) values of cells treated in (**a**). CI value < 1: synergistic, CI value = 1: additive, and CI > 1: antagonistic. The dotted line represents additivity (CI value = 1). FA: fraction affected; (**c**) The graphs show cellular viability of U251 and U87 cells treated with WEHI-539, THZ1, or the combination of both (*n* = 4); (**d**) Shown are the isobolograms of U251 and U87 cells treated with the indicated concentrations of ABT-199, THZ1 or the combination; (**e**) Shown is a graph with the CI (Combination Index) values of samples treated in (**d**); (**f**) The graphs show cellular viability of U251 and U87 cells treated with ABT-199, THZ1, or combination of both (*n* = 4); (**g**) The graphs show apoptotic cells of U87 and U251 cells treated with WEHI-539, THZ1, or the combination of both for 48h (*n* = 3); (**h,i**) Standard western blots of cell lysates of U251 and U87 cells treated with WEHI-539, THZ1, or the combination of both for 24h (FL: full length and CF: cleavage fragment). Actin is used as a loading control. The protein expression levels were quantified by using ImageJ (shown in cursive font). The full length of PARP protein expression was quantified. Shown are means and SD. ANOVA was used for statistical analysis. Uncropped blots are shown in Figure S10. ***p*<0.01, *****p*<0.001.


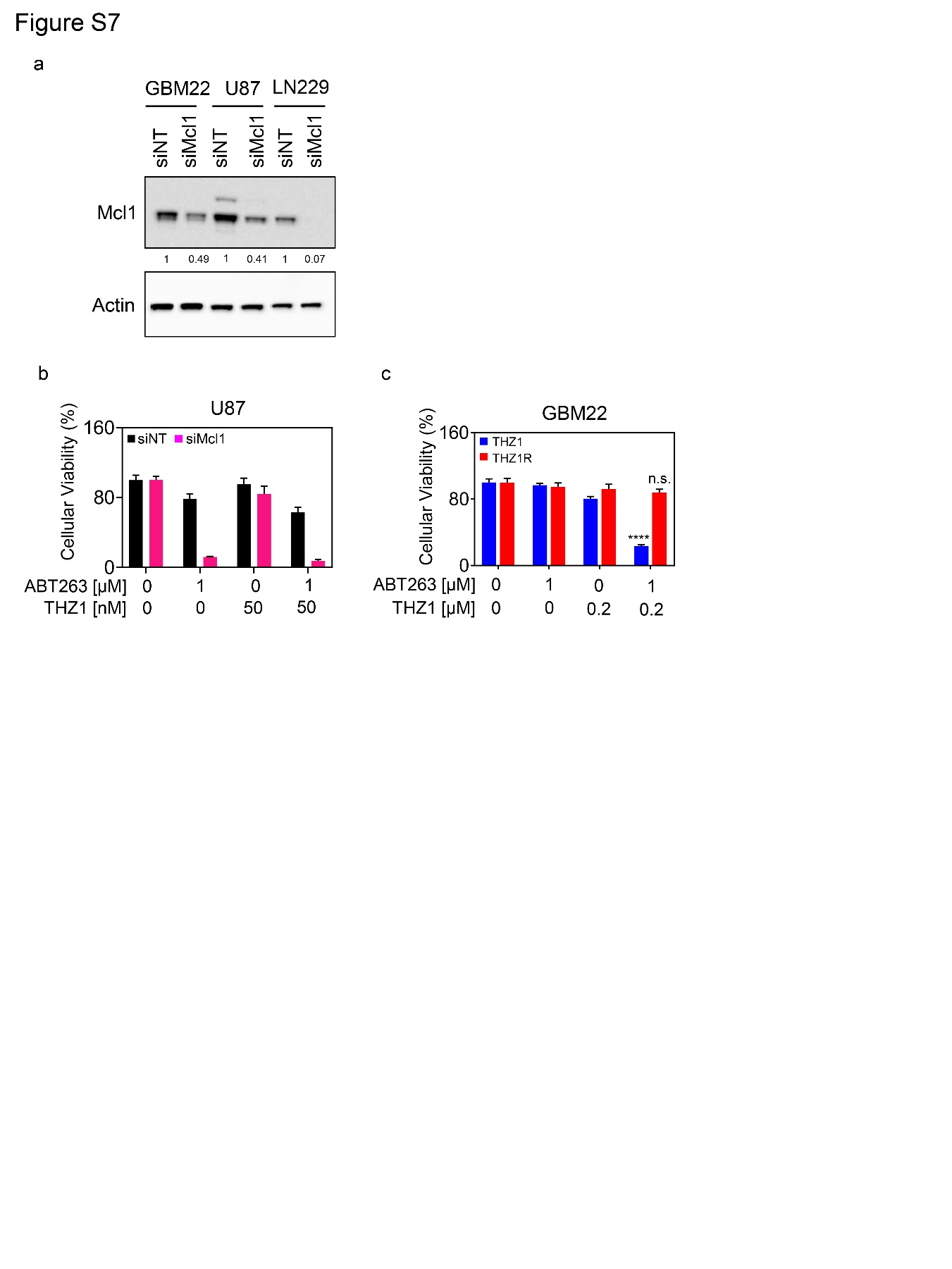


**Figure S7.** Down-regulation of Mcl-1 is involved in the combination treatment of ABT263 and THZ1 to exert its killing effects on GBM cells**.** (**a**) Standard western blots of cell lysates of GBM22, U87, and LN229 cells that were transfected with control (siNT) or specific Mcl-1 siRNAs. The protein expression level of Mcl1 was quantified by using ImageJ (shown in cursive font). Uncropped blots are shown in Figure S10; (**b**) Cellular viability of U87 cells transfected with control (siNT) or specific Mcl1 siRNAs and treated with ABT263, THZ1, or the combination of both; (**c**) Cellular viability of GBM22 cells treated with ABT263, THZ1/THZ1R, or the combination of both (n = 4); Shown are means and SD. ANOVA was used for statistical analysis. *****p* < 0.001, n.s.: not significant.

**
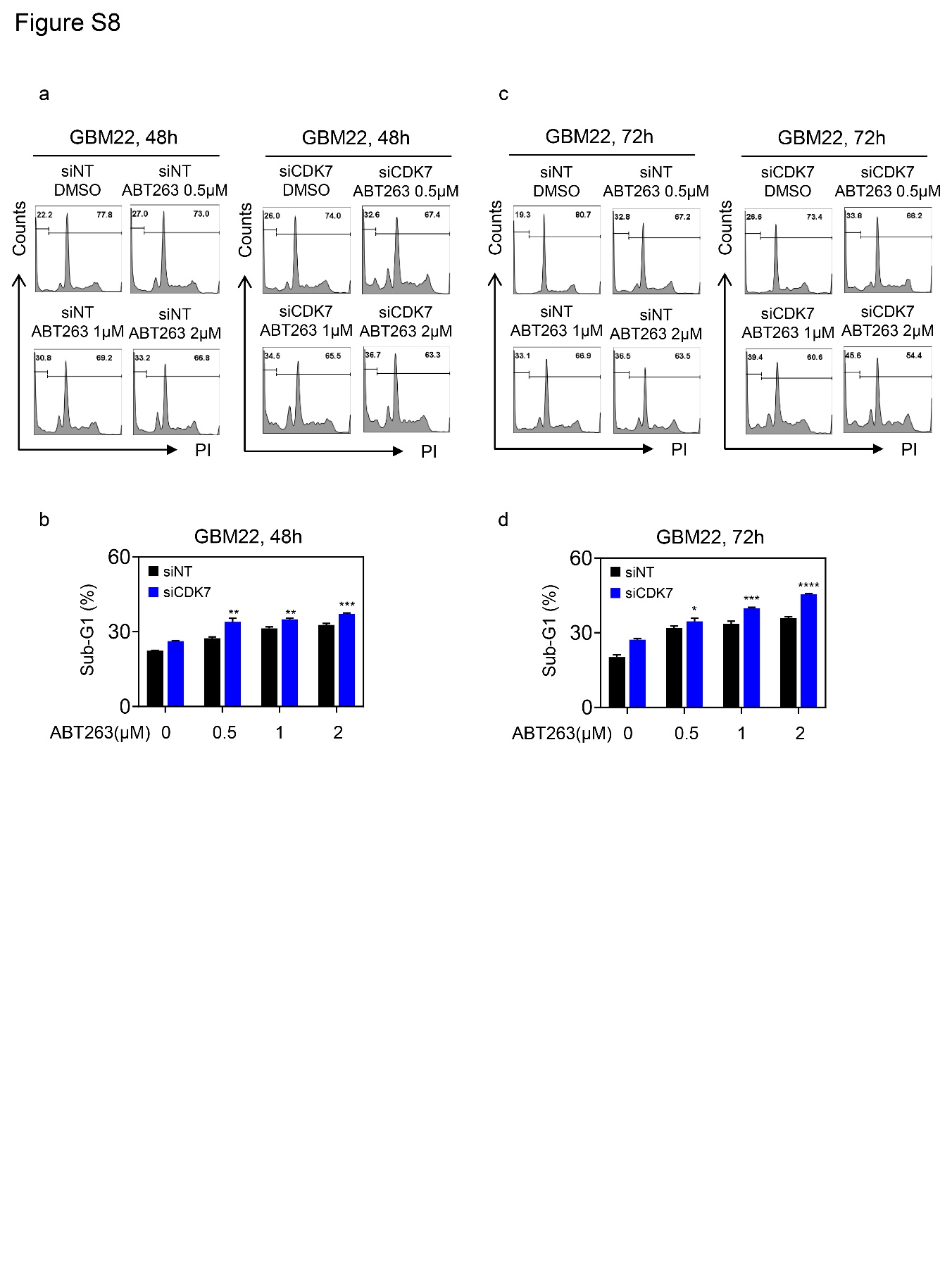
**

**Figure S8.** The sensitization effect by THZ1 to BH3-mimetic-mediated cell death involves additional targets other than the inhibition of CDK7. (**a**) Shown are representative flow plots of GBM22 cells transfected with control (siNT) or specific CDK7 siRNAs, treated with increasing concentration of ABT263 for 48 h, fixed and labeled with propidium iodide (PI) (for Sub-G1 fraction); (**b**) The graph shows the Sub-G1 fraction of GBM22 cells treated with ABT263, THZ1, or the combination of both for 48 h (*n* = 3); (**c**) Shown are representative flow plots of GBM22 cells transfected with control (siNT) or specific CDK7 siRNAs, treated with increasing concentration of ABT263 for 72 h, fixed and labeled with propidium iodide (PI) (for Sub-G1 fraction); (**d**) The graph shows the Sub-G1 fraction of GBM22 cells treated with ABT263, THZ1, or the combination of both for 72 h (*n* = 3); Shown are means and SD. Statistical significance was determined by two-tailed Student’s t-test. **p* < 0.05, ***p* < 0.01, ***/*****p* < 0.001.

**
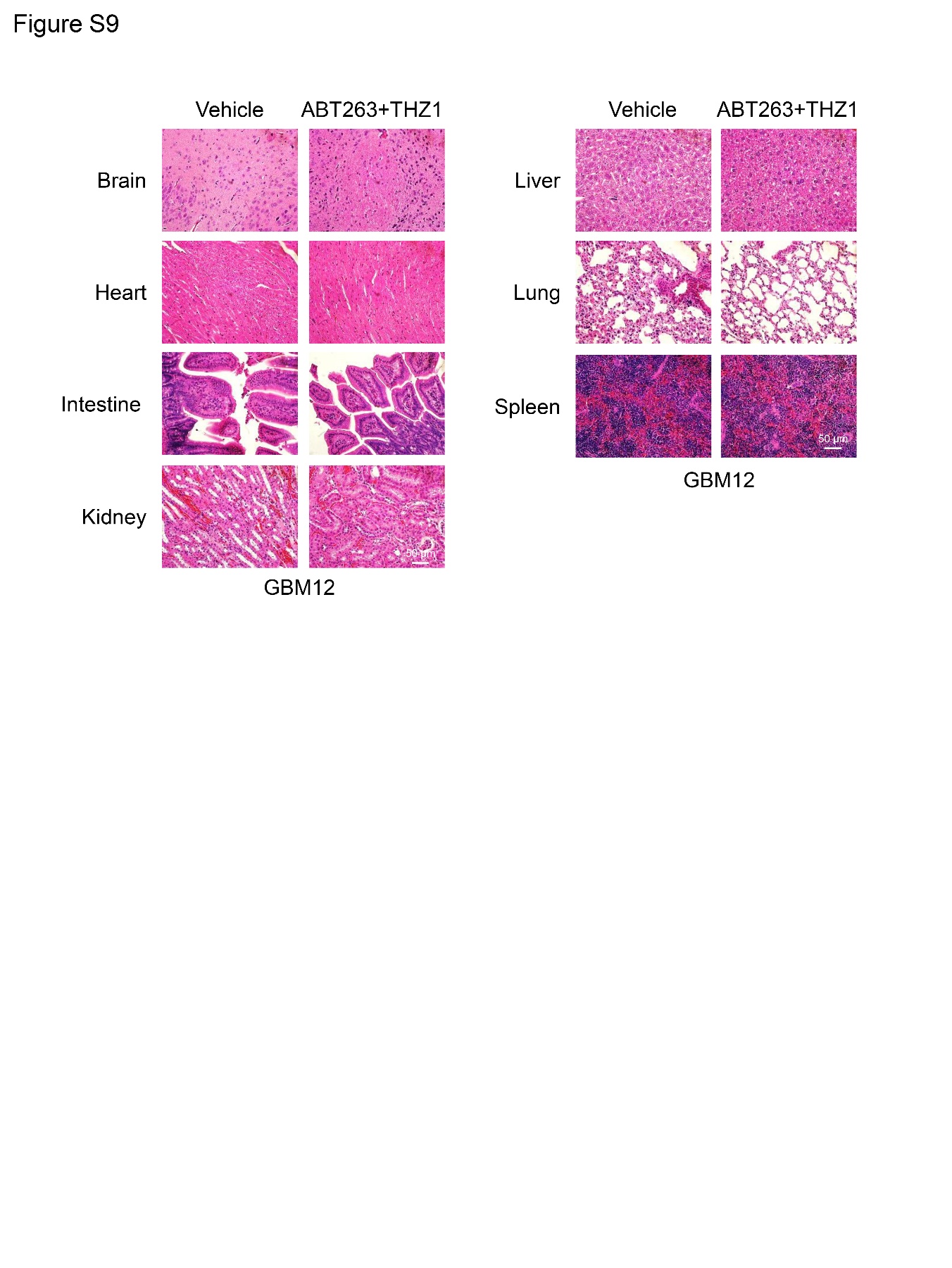
**

**Figure S9.** The combination treatment of ABT263 and THZ1 enhances anti-glioma activity in patient-derived xenograft in vivo without organ toxicity. GBM12 cells were implanted into the subcutis of immunocompromised Nu/Nu mice. Four randomly treatment groups, vehicle, ABT263 (75 mg/kg), THZ1 (10 mg/kg), and the combination treatment of both, were assigned after establishment of tumors. Mice were treated three times per week. At the end of the experiments, organs were harvested. Shown are the H&E staining of the vehicle and the combination treatment of ABT263 and THZ1 in different parenchymatous organs.

| 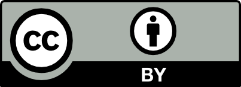 | © 2020 by the authors. Licensee MDPI, Basel, Switzerland. This article is an open access article distributed under the terms and conditions of the Creative Commons Attribution (CC BY) license (http://creativecommons.org/licenses/by/4.0/). |
| --- | --- |
