# Supplementary material for: Epigenetic Targeting of Mcl-1 Is Synthetically Lethal with Bcl-xL/Bcl-2 Inhibition in Model Systems of Glioblastoma
Source: Cancers (Basel). 2020 Aug 1;12(8):2137. doi: 10.3390/cancers12082137 (PMC7464325; doi:10.3390/cancers12082137)

Figure 2e

U87

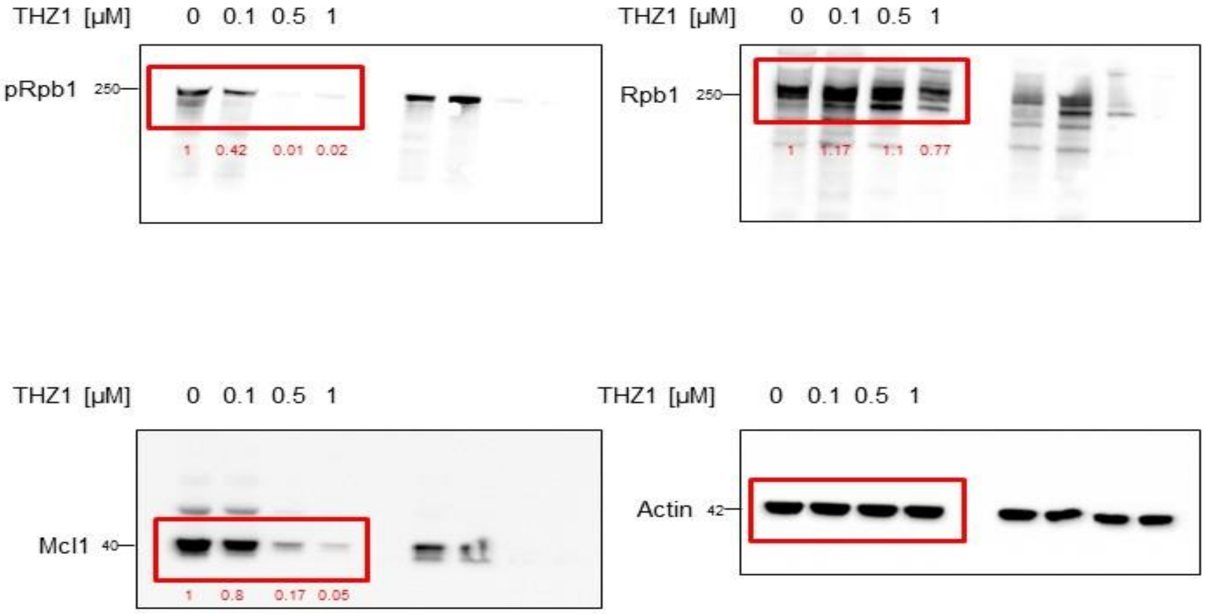

Figure 2e

GBM22

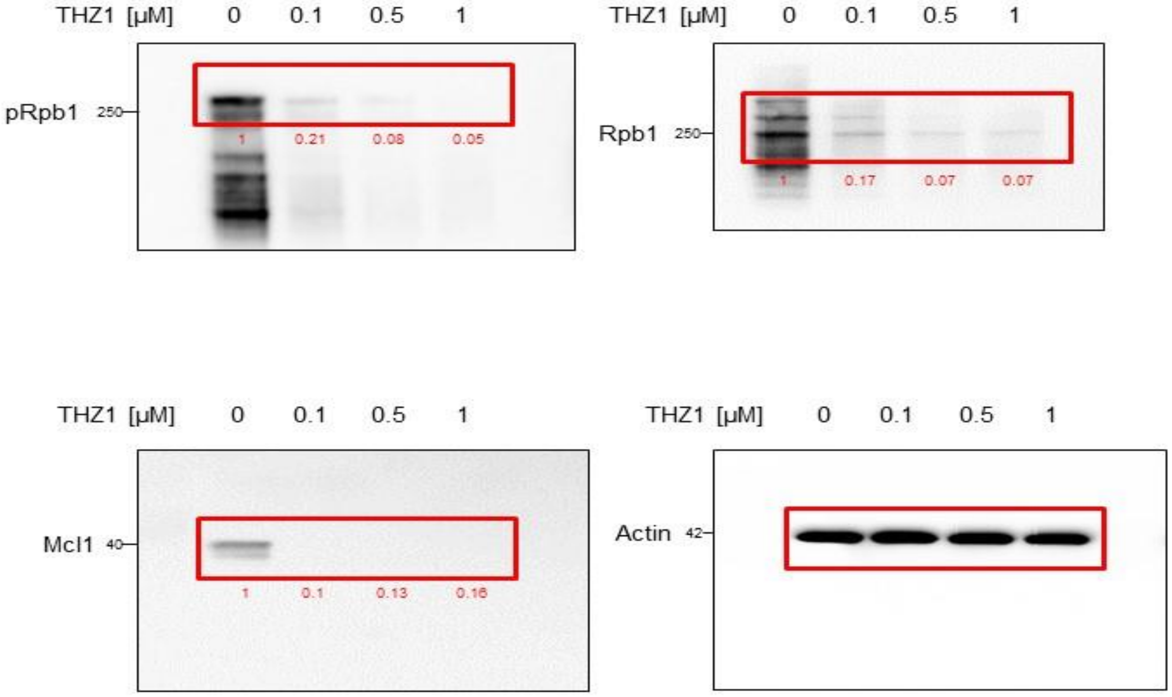

Figure 2f

U87, 24h

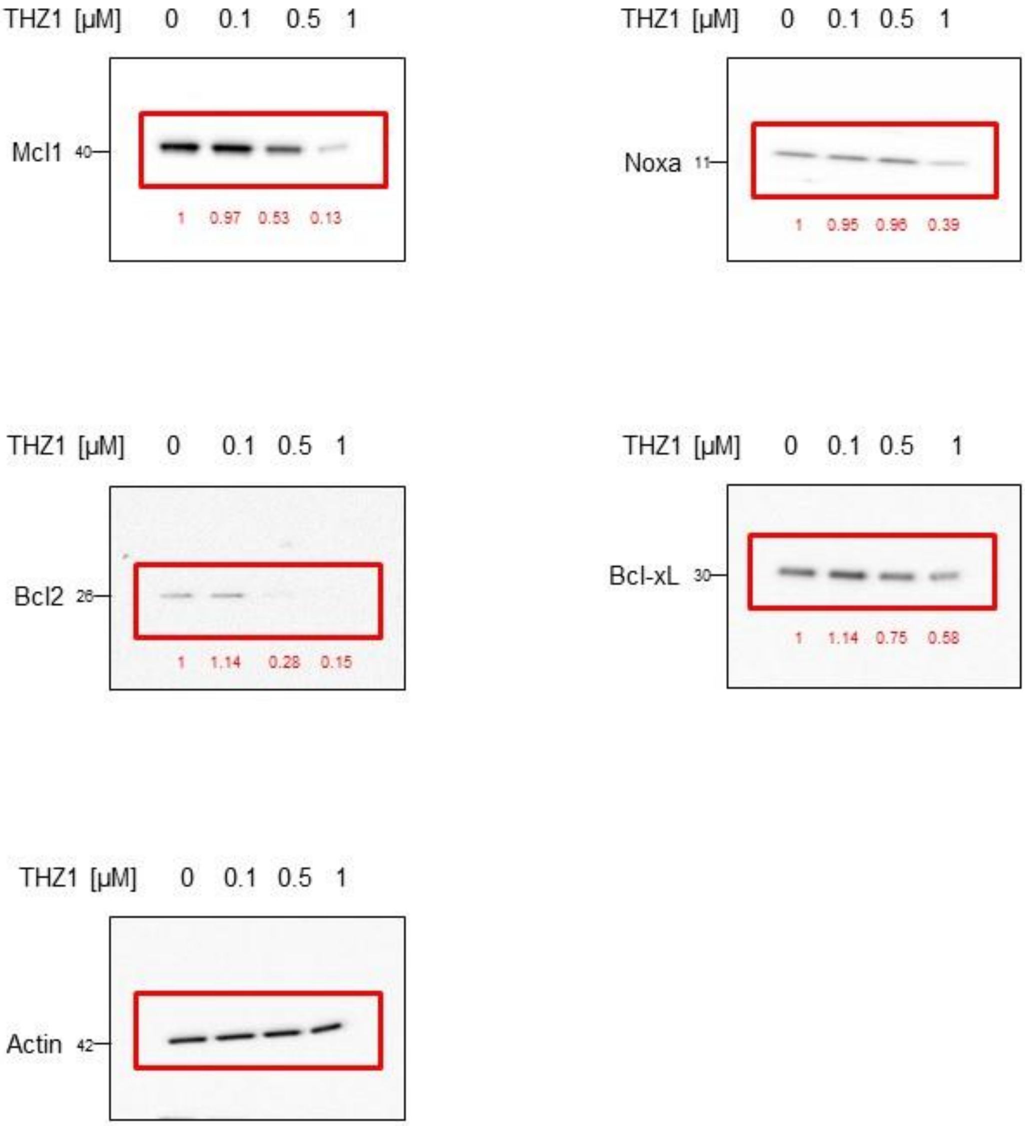

Figure 2f

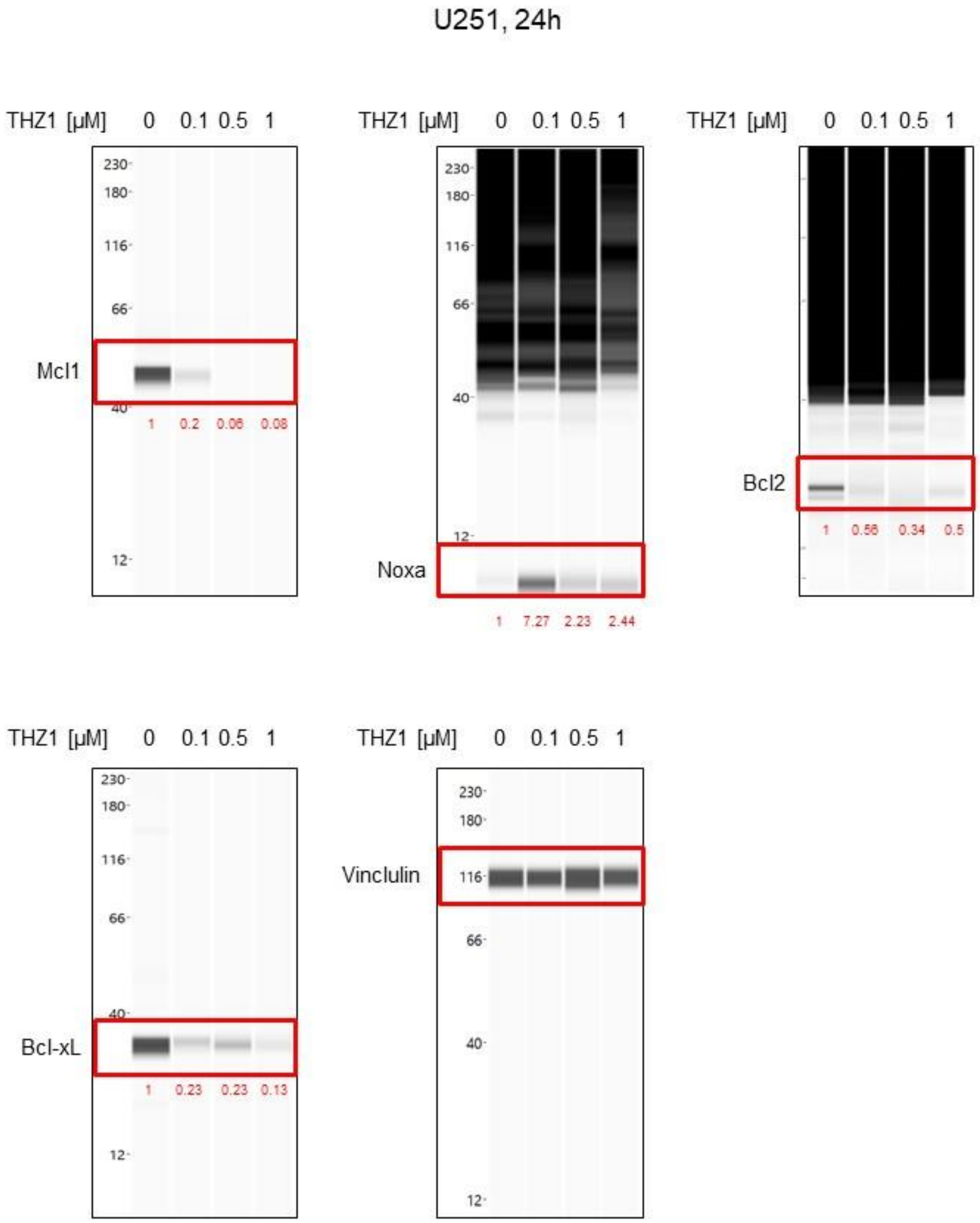

U251, 24h
THZ1 [μM] 0 0.1 0.5 1
230
180
116
66
40
12
Mcl1
1 0.2 0.06 0.08
THZ1 [μM] 0 0.1 0.5 1
Noxa
1 7.27 2.23 2.44
THZ1 [μM] 0 0.1 0.5 1
Bcl2
1 0.56 0.34 0.5
THZ1 [μM] 0 0.1 0.5 1
Bcl-xL
1 0.23 0.23 0.13
THZ1 [μM] 0 0.1 0.5 1
Vinclulin

Figure 2f

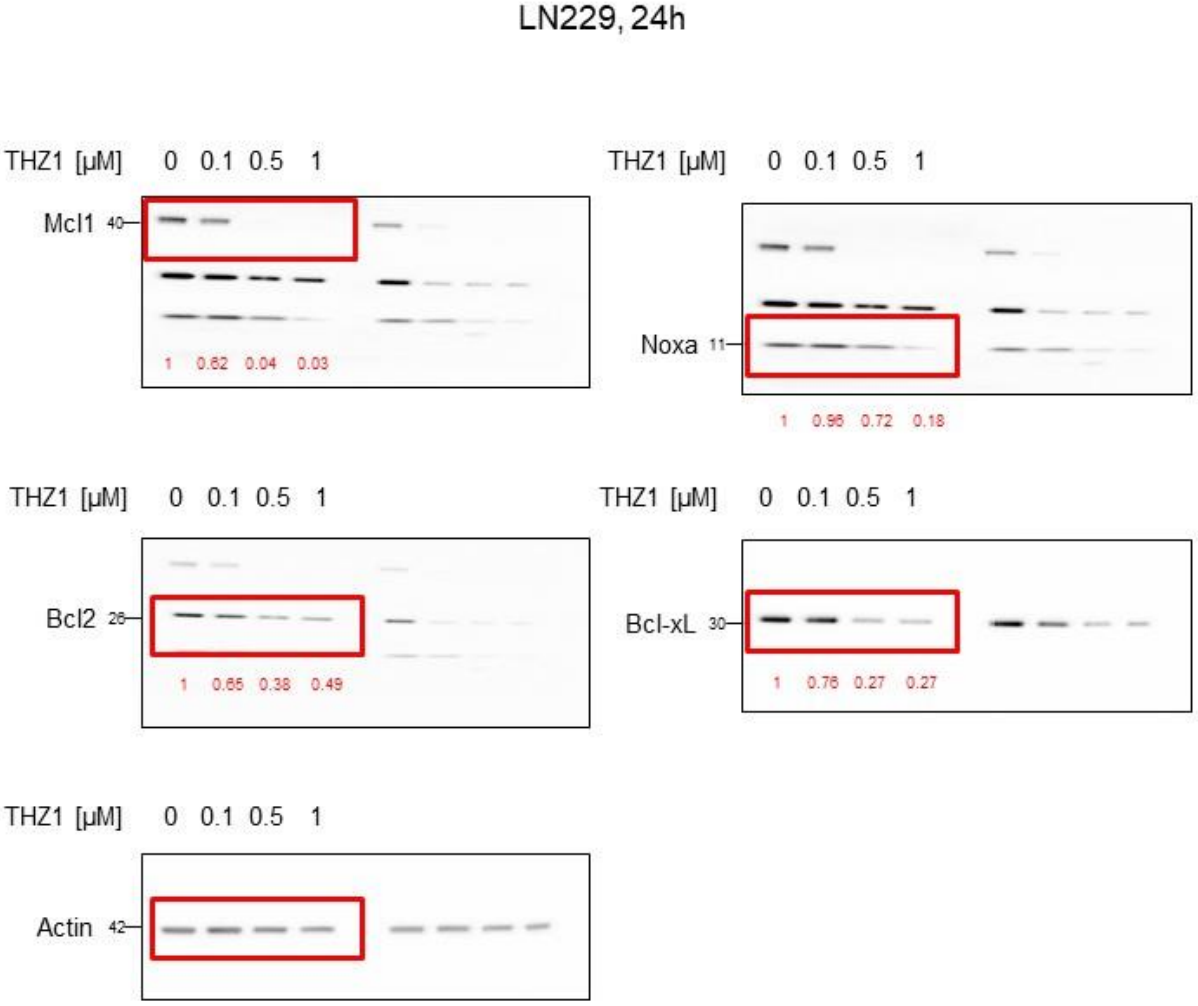
LN229, 24h
THZ1 [μM] 0 0.1 0.5 1
Mcl1 40
1 0.62 0.04 0.03
THZ1 [μM] 0 0.1 0.5 1
Noxa 11
1 0.96 0.72 0.18
THZ1 [μM] 0 0.1 0.5 1
Bcl2 26
1 0.65 0.38 0.49
THZ1 [μM] 0 0.1 0.5 1
Bcl-xL 30
1 0.76 0.27 0.27
THZ1 [μM] 0 0.1 0.5 1
Actin 42

Figure 2f

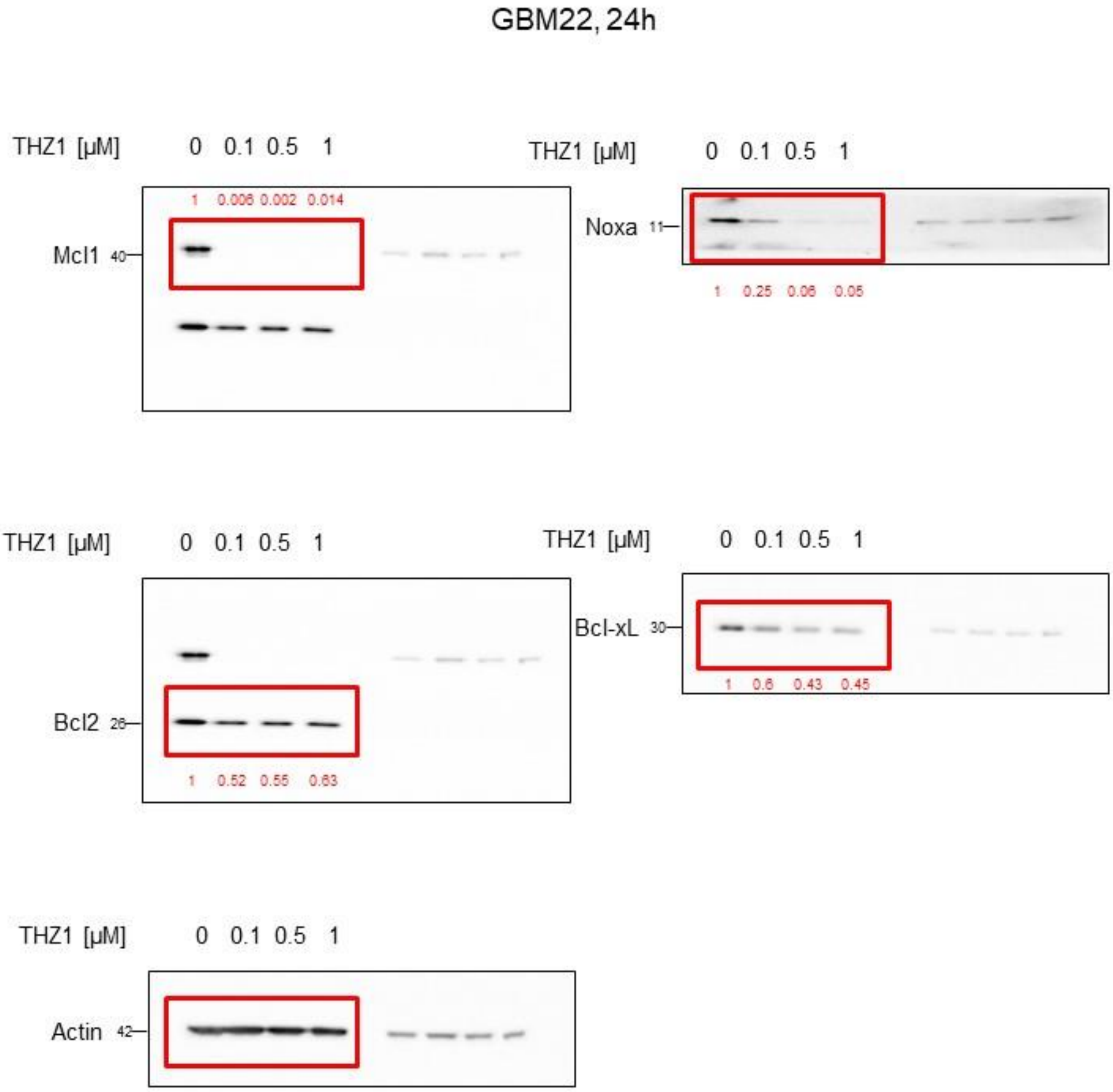
GBM22, 24h
THZ1 [μM] 0 0.1 0.5 1
1 0.006 0.002 0.014
Mcl1 40
THZ1 [μM] 0 0.1 0.5 1
Noxa 11
1 0.25 0.06 0.05
THZ1 [μM] 0 0.1 0.5 1
Bcl2 26
1 0.52 0.55 0.63
THZ1 [μM] 0 0.1 0.5 1
Bcl-xL 30
1 0.6 0.43 0.45
THZ1 [μM] 0 0.1 0.5 1
Actin 42

Figure 3d

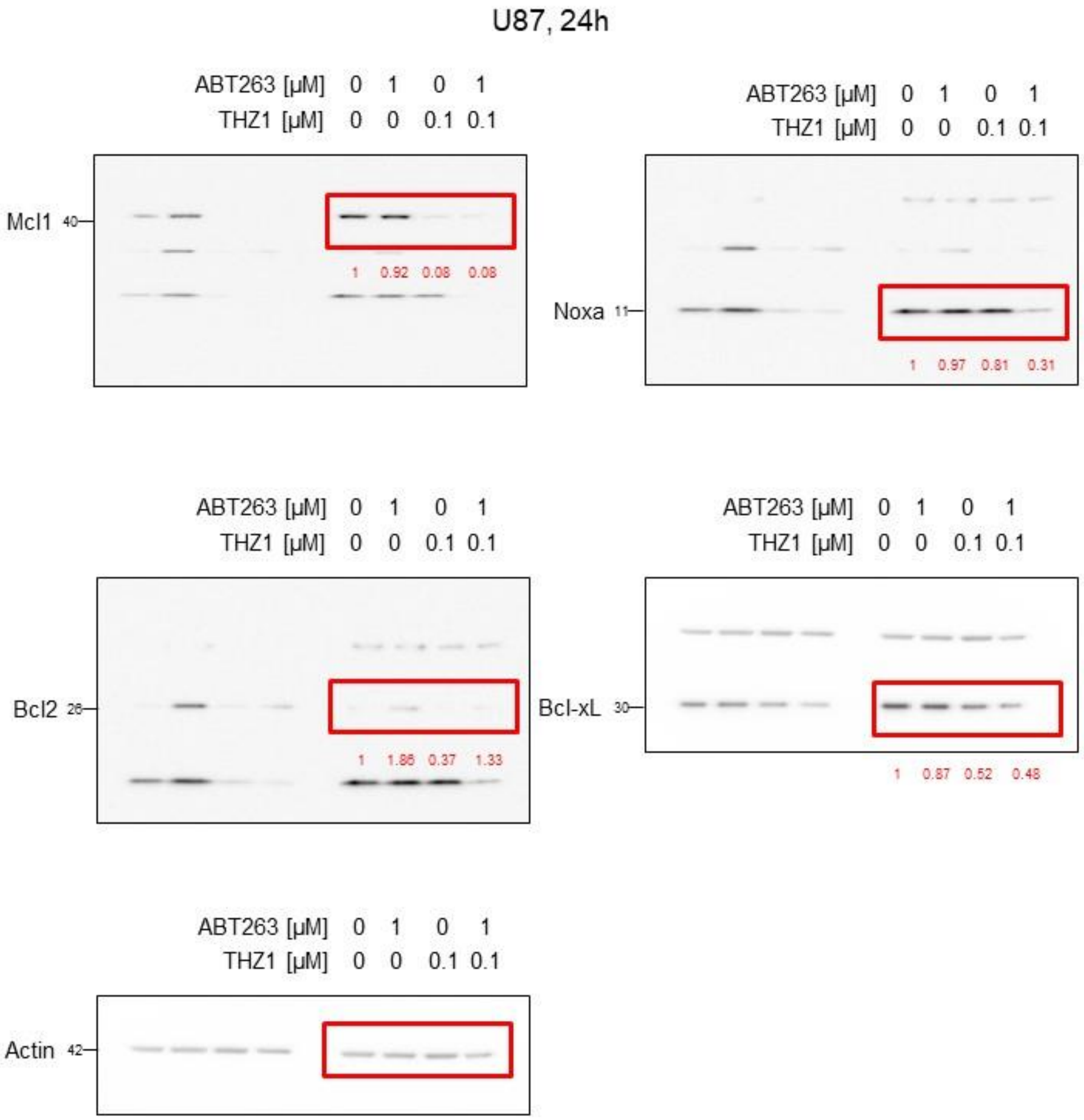
U87, 24h
ABT263 [µM] 0 1 0 1
THZ1 [µM] 0 0 0.1 0.1
Mcl1 40
1 0.92 0.08 0.08
ABT263 [µM] 0 1 0 1
THZ1 [µM] 0 0 0.1 0.1
Noxa 11
1 0.97 0.81 0.31
ABT263 [µM] 0 1 0 1
THZ1 [µM] 0 0 0.1 0.1
Bcl2 26
1 1.86 0.37 1.33
ABT263 [µM] 0 1 0 1
THZ1 [µM] 0 0 0.1 0.1
Bcl-xL 30
1 0.87 0.52 0.48
ABT263 [µM] 0 1 0 1
THZ1 [µM] 0 0 0.1 0.1
Actin 42

Figure 3d

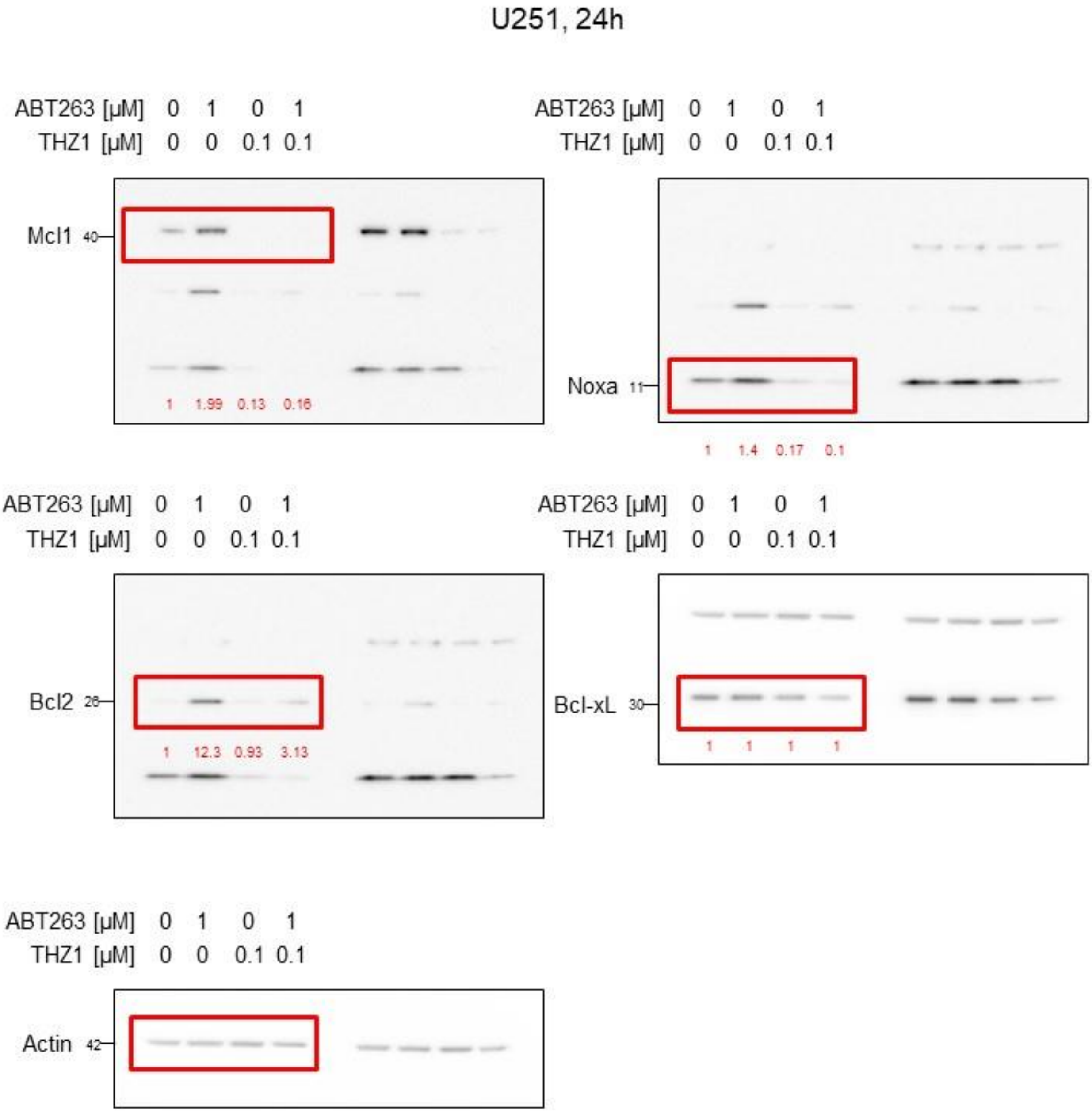

U251, 24h
ABT263 [µM] 0 1 0 1
THZ1 [µM] 0 0 0.1 0.1
Mcl1 40
1 1.99 0.13 0.16
ABT263 [µM] 0 1 0 1
THZ1 [µM] 0 0 0.1 0.1
Noxa 11
1 1.4 0.17 0.1
ABT263 [µM] 0 1 0 1
THZ1 [µM] 0 0 0.1 0.1
Bcl2 26
1 12.3 0.93 3.13
ABT263 [µM] 0 1 0 1
THZ1 [µM] 0 0 0.1 0.1
Bcl-xL 30
1 1 1 1
ABT263 [µM] 0 1 0 1
THZ1 [µM] 0 0 0.1 0.1
Actin 42

Figure 3d

LN229, 24h

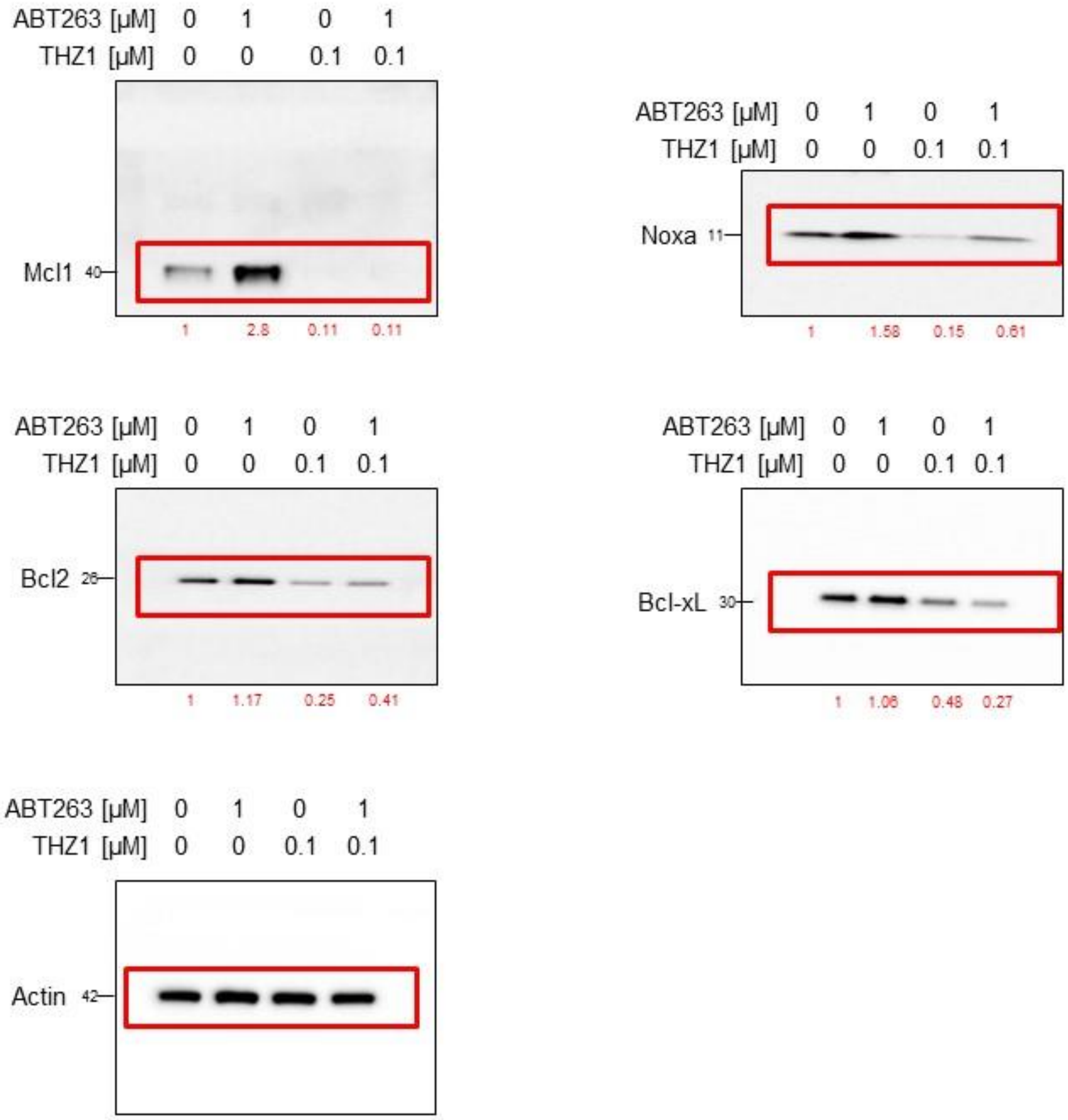

Figure 3d

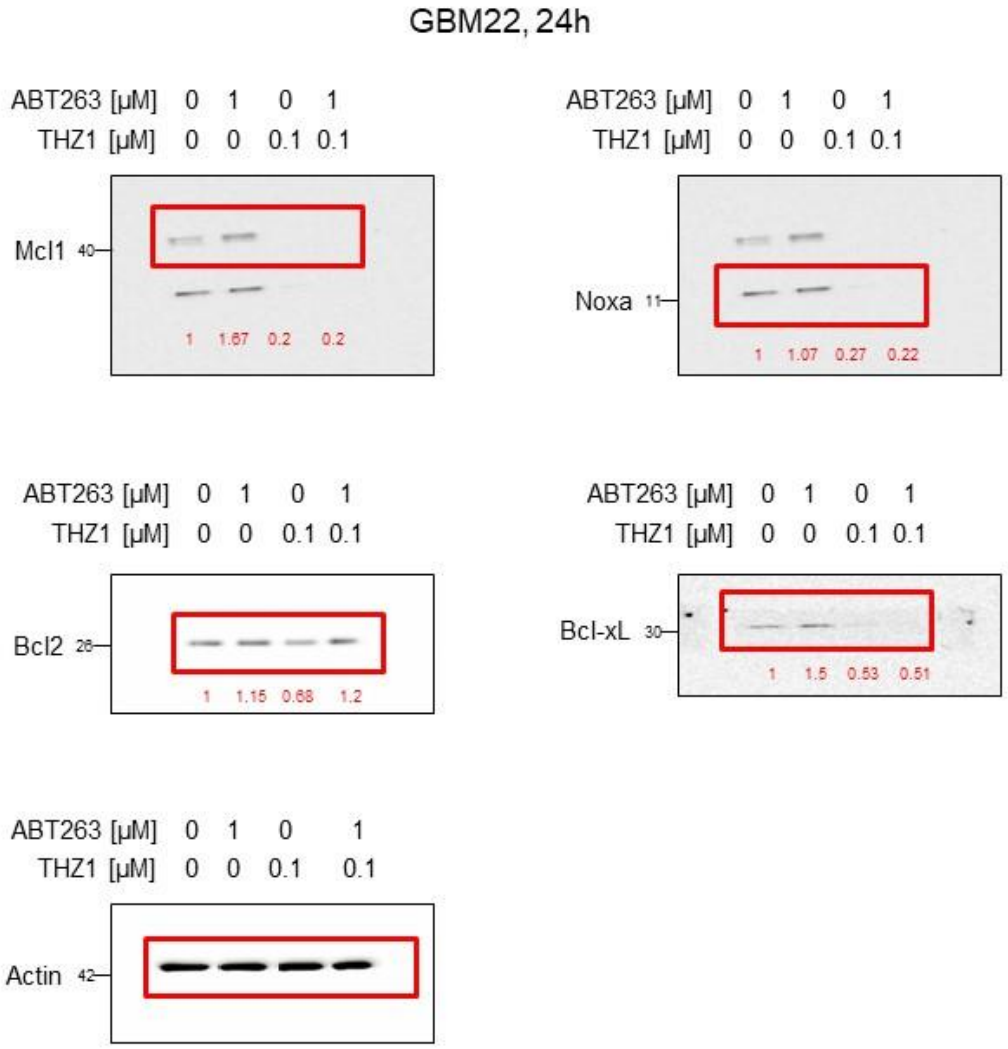
GBM22, 24h
ABT263 [µM] 0 1 0 1
THZ1 [µM] 0 0 0.1 0.1
Mcl1 40
1 1.67 0.2 0.2
ABT263 [µM] 0 1 0 1
THZ1 [µM] 0 0 0.1 0.1
Noxa 11
1 1.07 0.27 0.22
ABT263 [µM] 0 1 0 1
THZ1 [µM] 0 0 0.1 0.1
Bcl2 26
1 1.15 0.68 1.2
ABT263 [µM] 0 1 0 1
THZ1 [µM] 0 0 0.1 0.1
Bcl-xL 30
1 1.5 0.53 0.51
ABT263 [µM] 0 1 0 1
THZ1 [µM] 0 0 0.1 0.1
Actin 42

Figure 4e

U87, 24h

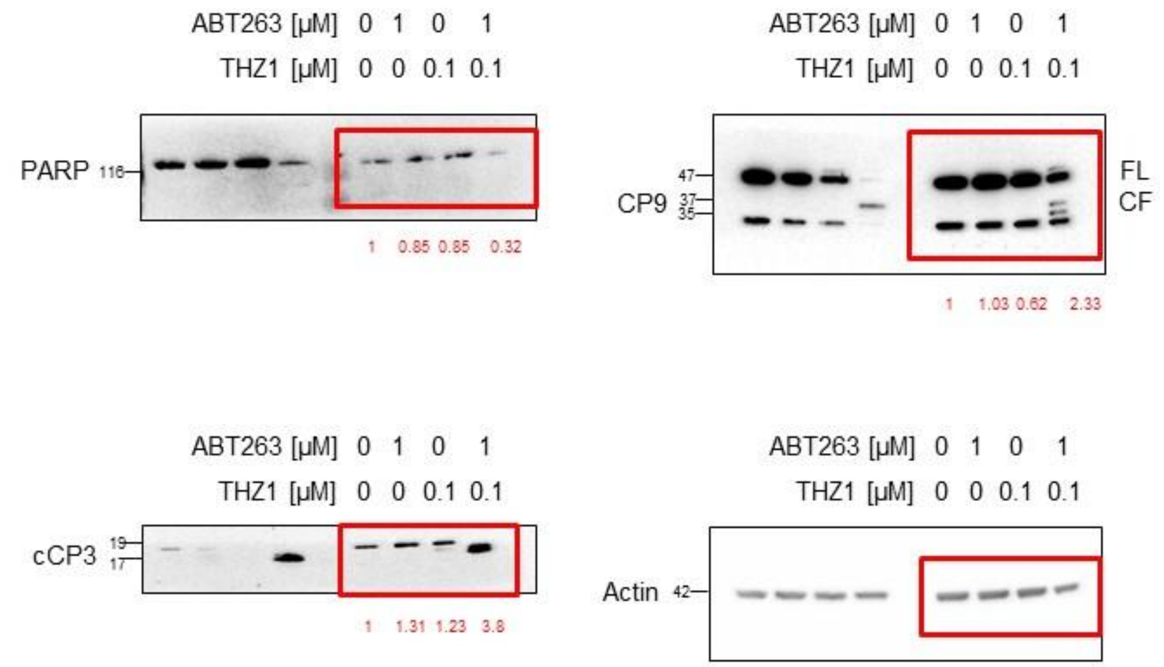

Figure 4e

U251, 24h

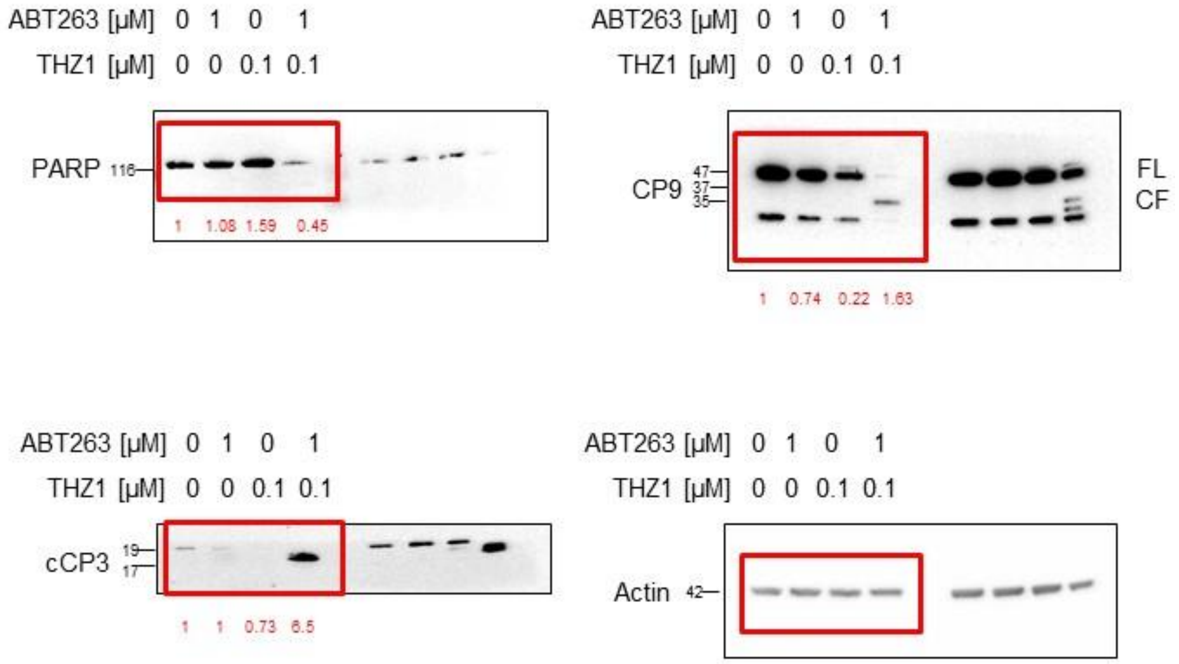

Figure 5c

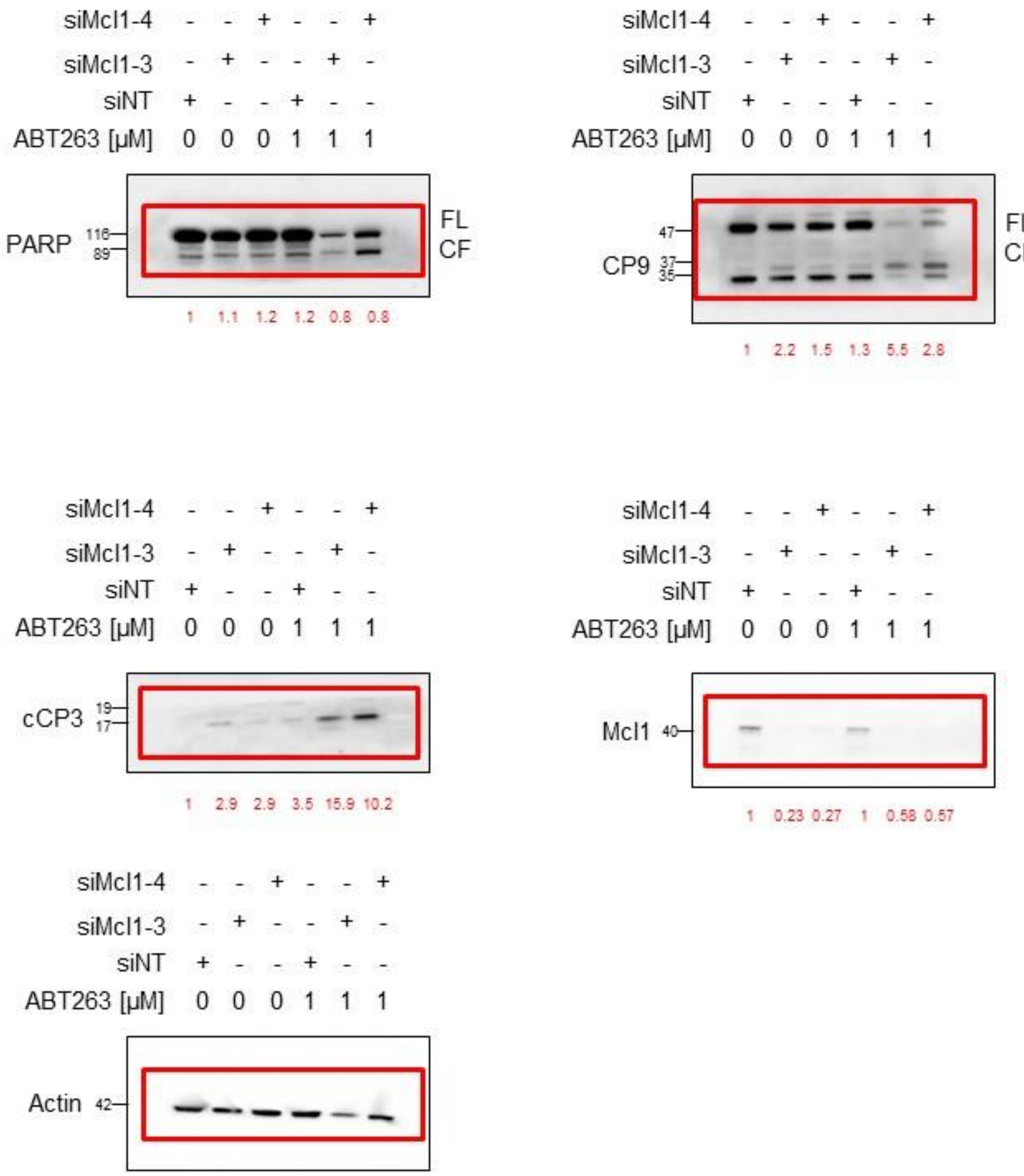
siMcl1-4 - - + - - +
siMcl1-3 - + - - + -
siNT + - - + - -
ABT263 [µM] 0 0 0 1 1 1
PARP
116
89
FL
CF
1 1.1 1.2 1.2 0.8 0.8
siMcl1-4 - - + - - +
siMcl1-3 - + - - + -
siNT + - - + - -
ABT263 [µM] 0 0 0 1 1 1
CP9
47
37
35
FL
CF
1 2.2 1.5 1.3 5.5 2.8
siMcl1-4 - - + - - +
siMcl1-3 - + - - + -
siNT + - - + - -
ABT263 [µM] 0 0 0 1 1 1
cCP3
19
17
1 2.9 2.9 3.5 15.9 10.2
siMcl1-4 - - + - - +
siMcl1-3 - + - - + -
siNT + - - + - -
ABT263 [µM] 0 0 0 1 1 1
Mcl1
40
1 0.23 0.27 1 0.58 0.57
siMcl1-4 - - + - - +
siMcl1-3 - + - - + -
siNT + - - + - -
ABT263 [µM] 0 0 0 1 1 1
Actin
42

Figure S1d

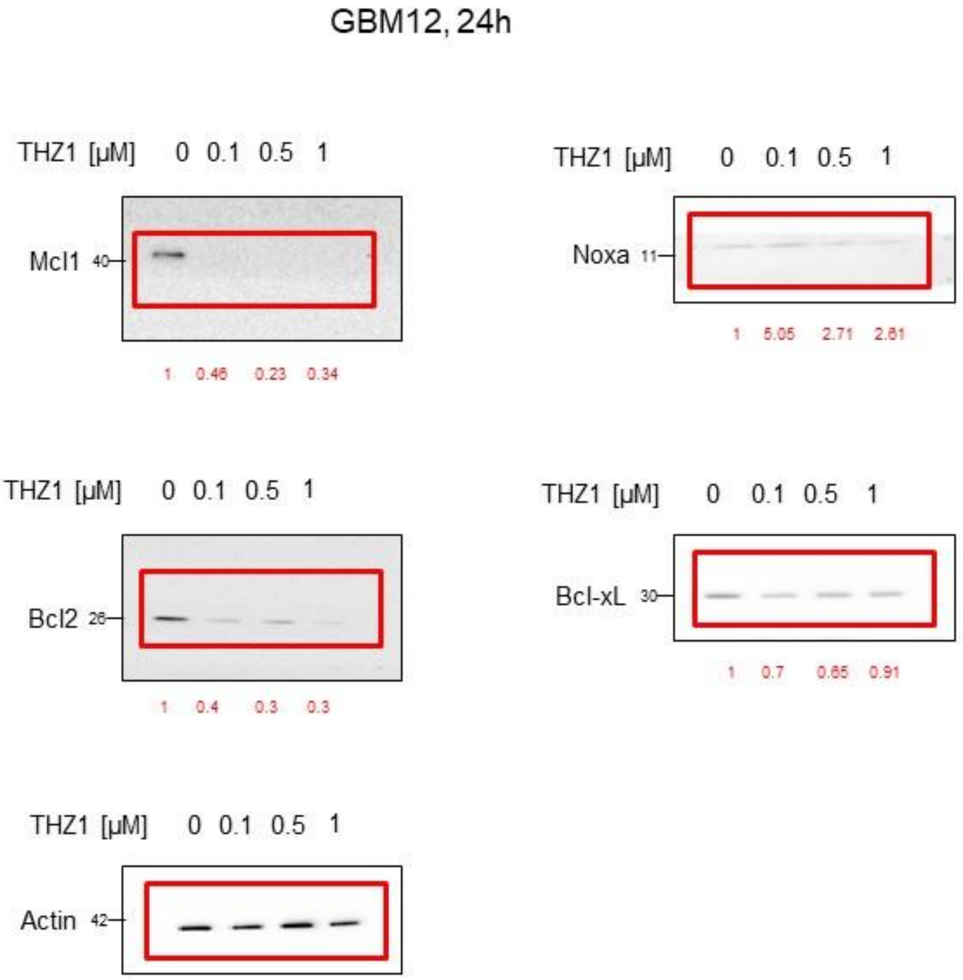
GBM12, 24h
THZ1 [µM] 0 0.1 0.5 1
Mcl1 40
1 0.46 0.23 0.34
THZ1 [µM] 0 0.1 0.5 1
Noxa 11
1 5.05 2.71 2.61
THZ1 [µM] 0 0.1 0.5 1
Bcl2 26
1 0.4 0.3 0.3
THZ1 [µM] 0 0.1 0.5 1
Bcl-xL 30
1 0.7 0.65 0.91
THZ1 [µM] 0 0.1 0.5 1
Actin 42

Figure S1d

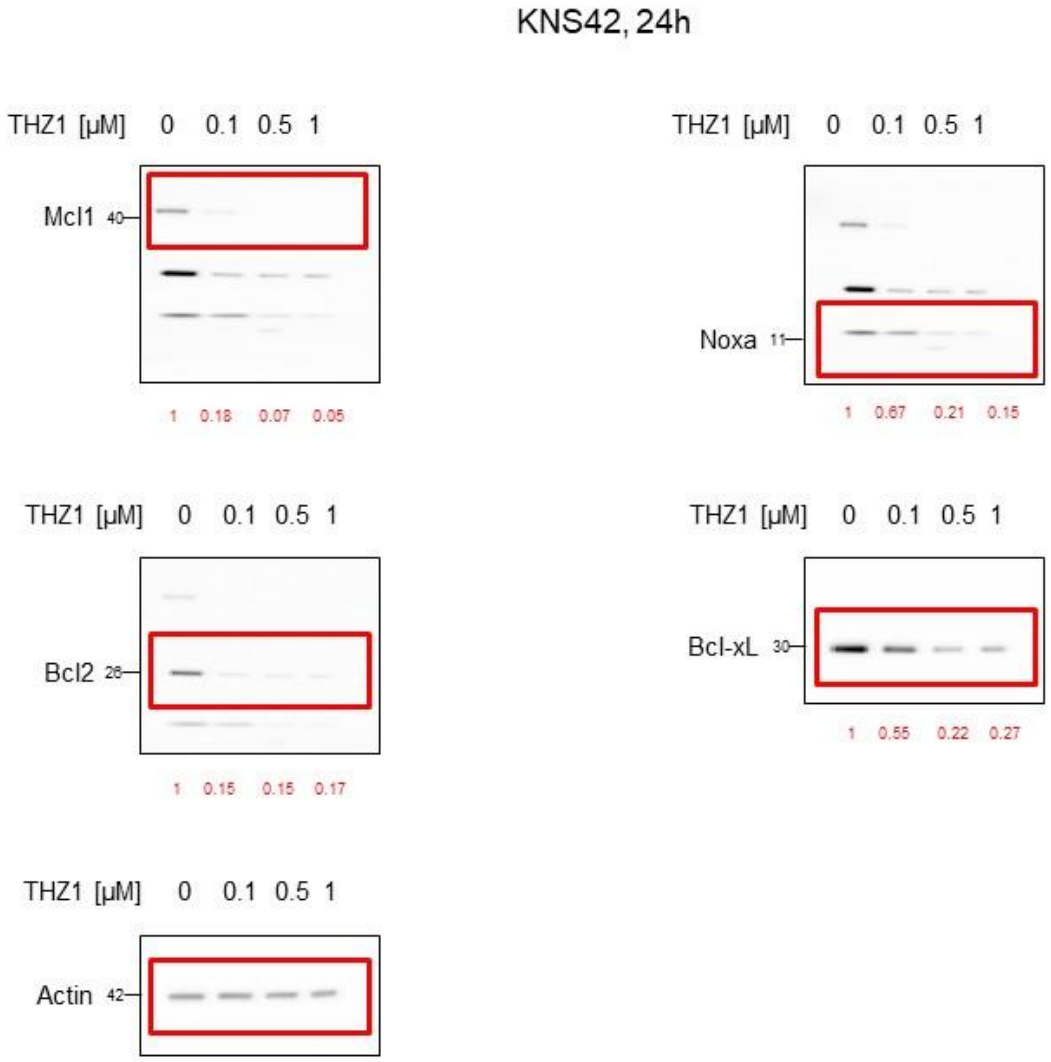
KNS42, 24h
THZ1 [µM] 0 0.1 0.5 1
Mcl1 40
1 0.18 0.07 0.05
THZ1 [µM] 0 0.1 0.5 1
Noxa 11
1 0.67 0.21 0.15
THZ1 [µM] 0 0.1 0.5 1
Bcl2 26
1 0.15 0.15 0.17
THZ1 [µM] 0 0.1 0.5 1
Bcl-xL 30
1 0.55 0.22 0.27
THZ1 [µM] 0 0.1 0.5 1
Actin 42

Figure S5d

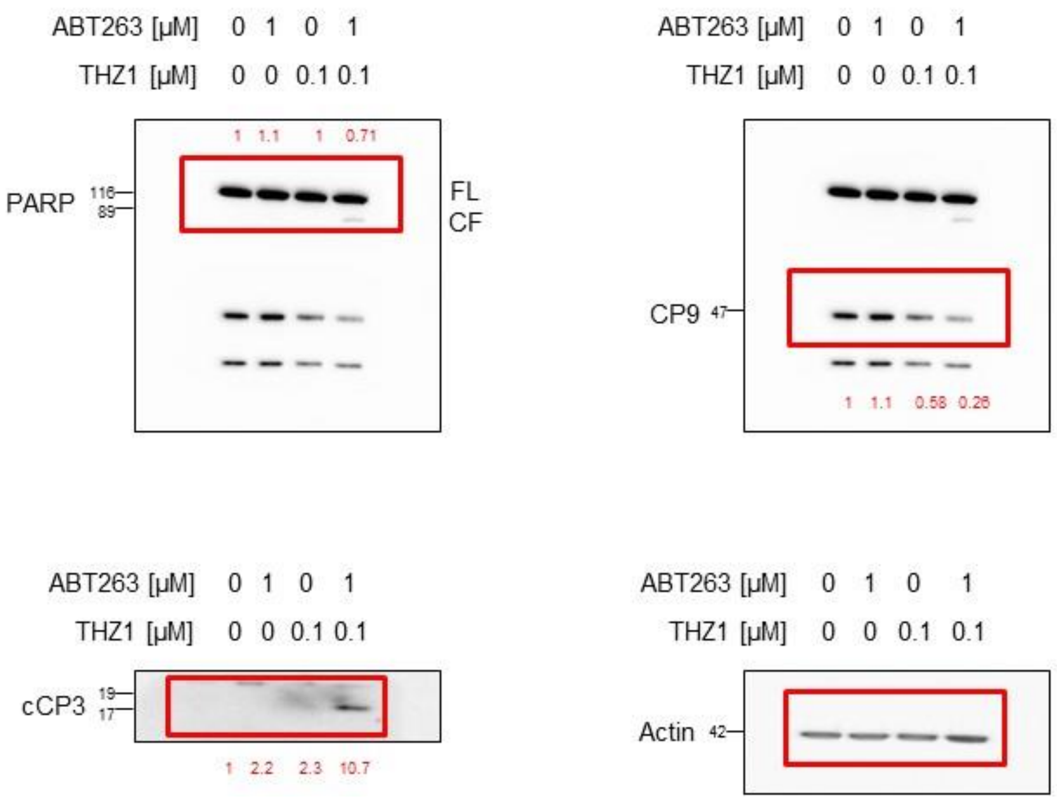
ABT263 [μM] 0 1 0 1
THZ1 [μM] 0 0 0.1 0.1
1 1.1 1 0.71
PARP
116
89
FL
CF
ABT263 [μM] 0 1 0 1
THZ1 [μM] 0 0 0.1 0.1
CP9
47
1 1.1 0.58 0.26
ABT263 [μM] 0 1 0 1
THZ1 [μM] 0 0 0.1 0.1
cCP3
19
17
1 2.2 2.3 10.7
ABT263 [μM] 0 1 0 1
THZ1 [μM] 0 0 0.1 0.1
Actin
42

Figure S6h

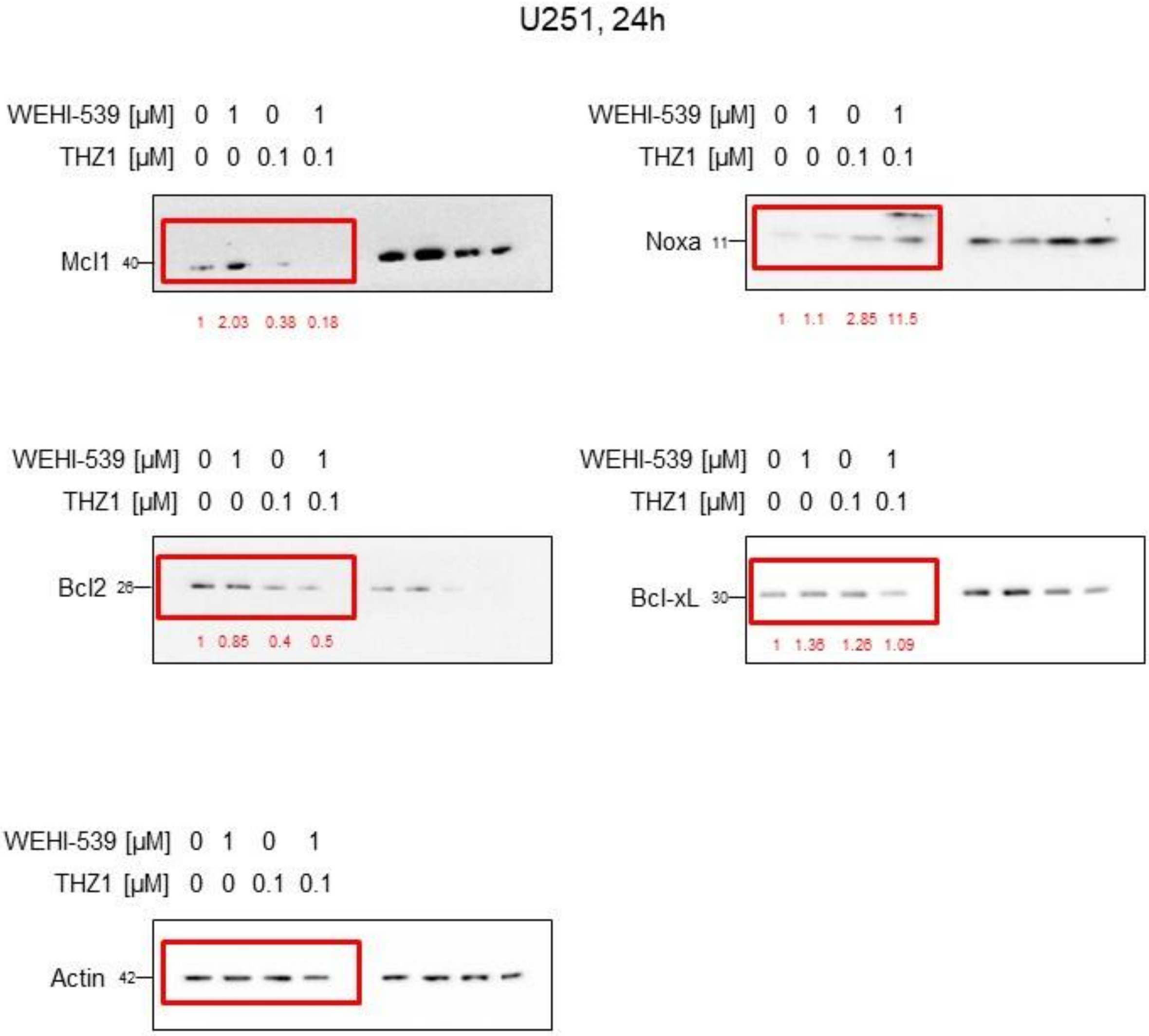
U251, 24h
WEHI-539 [µM] 0 1 0 1
THZ1 [µM] 0 0 0.1 0.1
Mcl1 40
1 2.03 0.38 0.18
WEHI-539 [µM] 0 1 0 1
THZ1 [µM] 0 0 0.1 0.1
Noxa 11
1 1.1 2.85 11.5
WEHI-539 [µM] 0 1 0 1
THZ1 [µM] 0 0 0.1 0.1
Bcl2 26
1 0.85 0.4 0.5
WEHI-539 [µM] 0 1 0 1
THZ1 [µM] 0 0 0.1 0.1
Bcl-xL 30
1 1.36 1.26 1.09
WEHI-539 [µM] 0 1 0 1
THZ1 [µM] 0 0 0.1 0.1
Actin 42

Figure S6h

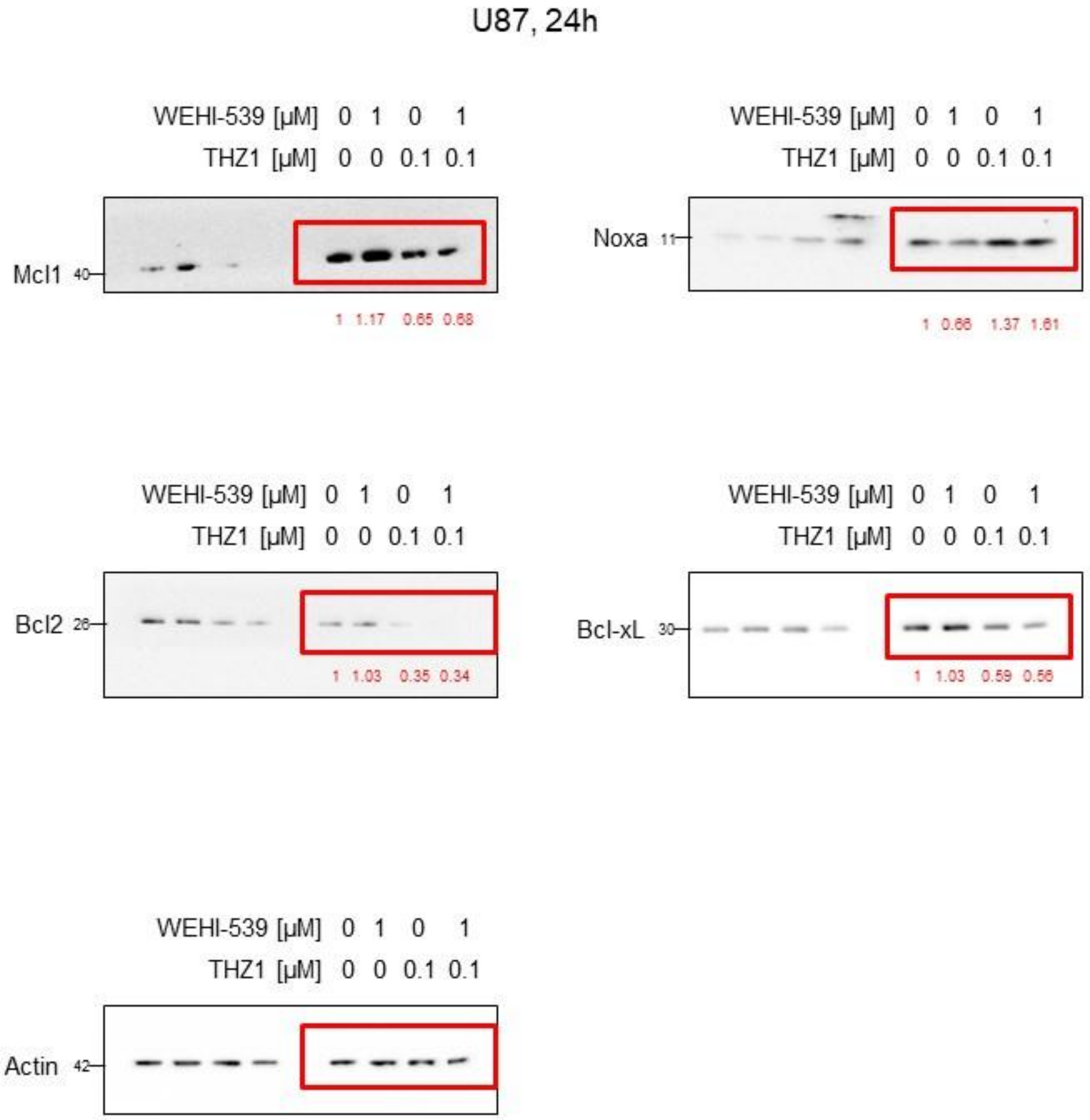
U87, 24h
WEHI-539 [µM] 0 1 0 1
THZ1 [µM] 0 0 0.1 0.1
Mcl1 40
1 1.17 0.65 0.68
WEHI-539 [µM] 0 1 0 1
THZ1 [µM] 0 0 0.1 0.1
Noxa 11
1 0.66 1.37 1.61
WEHI-539 [µM] 0 1 0 1
THZ1 [µM] 0 0 0.1 0.1
Bcl2 26
1 1.03 0.35 0.34
WEHI-539 [µM] 0 1 0 1
THZ1 [µM] 0 0 0.1 0.1
Bcl-xL 30
1 1.03 0.59 0.56
WEHI-539 [µM] 0 1 0 1
THZ1 [µM] 0 0 0.1 0.1
Actin 42

Figure S6i

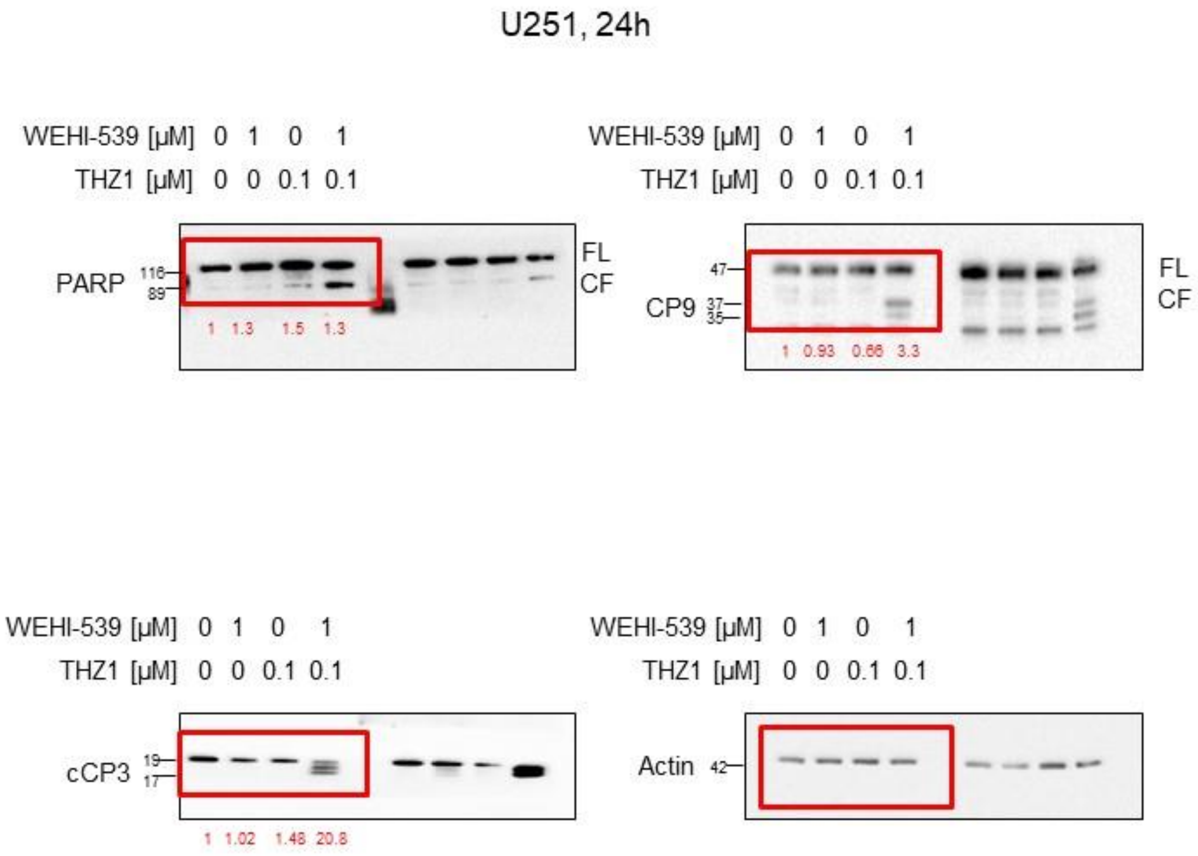
U251, 24h
WEHI-539 [µM] 0 1 0 1
THZ1 [µM] 0 0 0.1 0.1
PARP
116
89
FL
CF
1 1.3 1.5 1.3
WEHI-539 [µM] 0 1 0 1
THZ1 [µM] 0 0 0.1 0.1
CP9
47
37
35
FL
CF
1 0.93 0.66 3.3
WEHI-539 [µM] 0 1 0 1
THZ1 [µM] 0 0 0.1 0.1
cCP3
19
17
1 1.02 1.48 20.8
WEHI-539 [µM] 0 1 0 1
THZ1 [µM] 0 0 0.1 0.1
Actin
42

Figure S6i

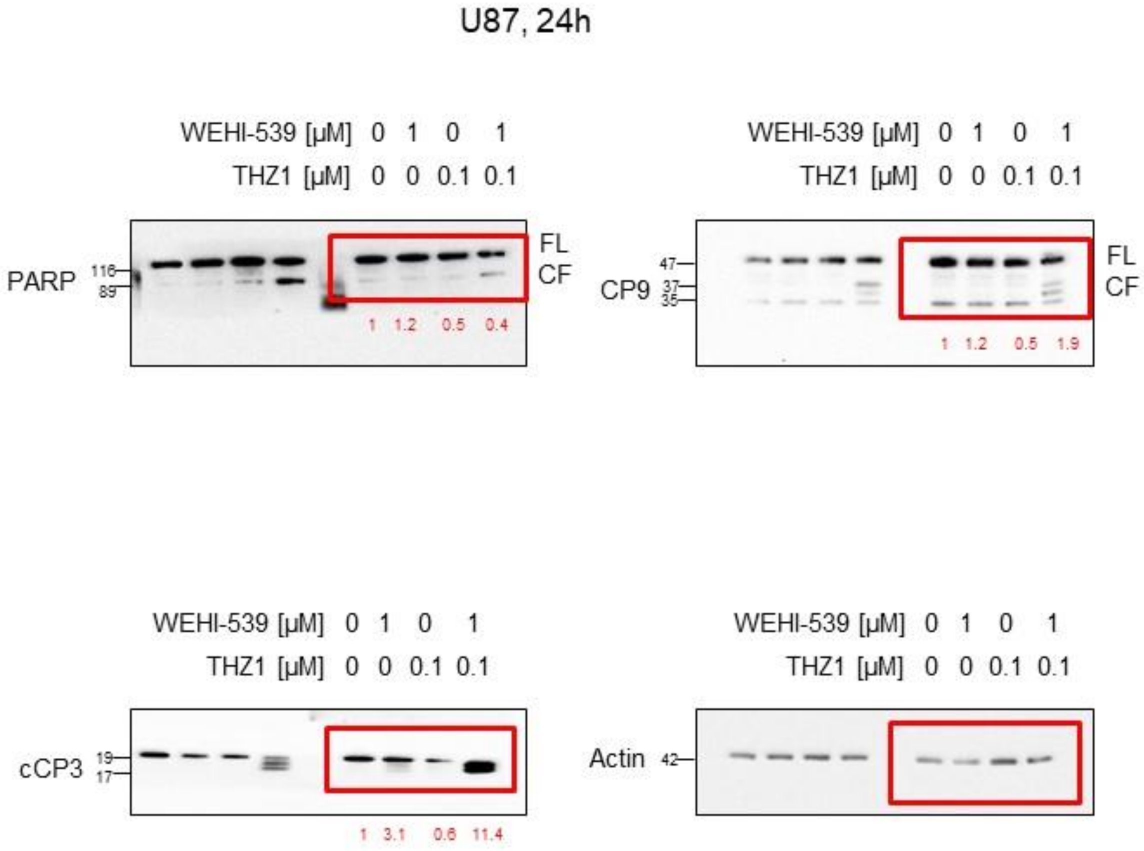

Figure S7a

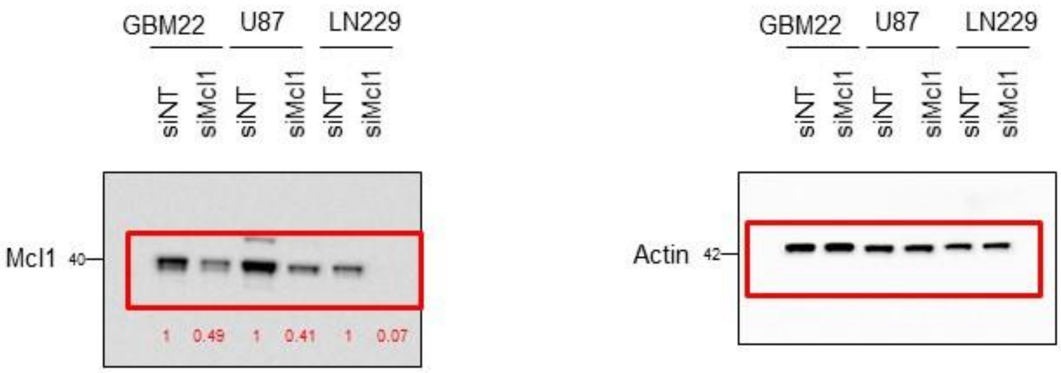

Supplement: Supplementary file 1 [file cancers-12-02137-s001.zip › cancers-859599-supplementary final/Figure S10-Uncropped Western Blot Figures.pdf]
